# The efficacy of the theory of planned behaviour and value-belief-norm theory for predicting young Chinese intention to choose green hotels

**DOI:** 10.1038/s41598-025-99447-1

**Published:** 2025-04-24

**Authors:** Qi Zhang, Philip Pong Weng Wong, Lei Wang

**Affiliations:** 1 Business School, Xuzhou University of Technology, Xuzhou, China; 2 School of Hospitality and Service Management, Sunway University, Petaling Jaya, Malaysia; 3 Faculty of Hospitality and Tourism, Xuzhou University of Technology, Xuzhou, China

**Keywords:** Collectivistic value, Intention to visit green hotels, Theory of planned behaviour, Value-belief-norm theory, Human behaviour, Psychology, Environmental social sciences

## Abstract

Although hotels have begun to pay more attention to green practices in providing products and services to satisfy guests’ green needs, the percentage of guests visiting green hotels is still low. Researchers undervalue the connection between consumers’ values, attitudes, norms, and intentions to stay at green hotels. Recently, scholars suggested that the attitude-intention gap can be closed by combining the theory of planned behaviour and the value-belief-norm theory. Therefore, this study examined the interrelationships among various constructs based on both theories. A total of 406 university students from Xuzhou City, China were employed quantitatively, utilising a purposive sample technique to test hypotheses using the Statistical Package for the Social Sciences and structural equation modelling. The results demonstrated that altruistic value, biospheric value, and collectivistic value positively influenced ecocentric attitude respectively. Implicit attitude was positively influenced by biospheric value, collectivistic value, and ecocentric attitude. Meanwhile, ecocentric attitude positively influenced awareness of consequence, subsequently ascription of responsibility, then the personal norm, and finally intention. Furthermore, implicit attitude, subjective norm, and perceived behavioural control positively influenced intention separately while subjective norm favourably influenced implicit attitude, perceived behavioural control, and personal norm respectively. The findings of this study would significantly contribute to a deeper understanding of consumers’ green hotel visits based on a combined theoretical framework from a developing country with a highly collectivistic value orientation, which benefits the growth of the green hotel industry and other key stakeholders.

**Supplementary Information:**

The online version contains supplementary material available at 10.1038/s41598-025-99447-1.

## Introduction

Future consumption patterns will be determined by consumers’ ecologically sensitive purchase decisions^1^. Consumers are beginning to express favourability towards the products and services provided by businesses that opt to incorporate environmentally friendly practices into their operations^2^. They now are price-conscious and willing to pay a premium for environmentally friendly products^3,4^. Businesses with environmentally conscious value propositions are more likely to survive the fiercely competitive market^5^. One method to ensure sustainability is through the advancement of smart technology, manufacturing, and material production^6^. In many nations, the hotel industry has emerged as one of the primary drivers of economic growth^7^. However, research has shown that the hotel industry significantly contributes to global warming^8^, and a significant portion of the adverse environmental effects hotels cause are related to their excessive use of energy and natural resources^9^. Pollution that finds its way into the air, water, or soil consequently raises environmental awareness and concerns^10^. Because of increased consumer concerns about those issues and expectations that hotels raise their green consciousness^11^, the hospitality sector is currently implementing more ecologically friendly practices^12^.

The concept of “Green Hotel” is defined as lodging establishments that use a range of environmentally friendly techniques to reduce waste and expenses associated with operations (e.g., using water, energy, natural resources, natural lighting, natural ventilation, and recyclable-based materials for furnishings)^13^. Hoteliers believe that sustainable practices enhance living standards, augment the calibre of products and services, and aid in their ability to remain competitive in a world that is changing rapidly^14^. Besides that, as guests ultimately decide whether or not to use green practices, the implementation of a green hotel strategy mostly depends on guest demand^15^. More proof of this can be found in recent research indicating that guests prefer eco-friendly hotels to more conventional hotels^16,17^.

Despite numerous consumers expressing a preference for green hotels^7^, the escalating intricacies of sustainability and persistent challenges in formulating innovative marketing strategies for green hotels^15^ appear to undermine their attractiveness to tourists^3^. Hotels have redirected their focus towards implementing sustainable practices to enhance competitiveness^6^, prompted by travellers’ heightened awareness of environmental concerns and favourable attitudes towards green accommodations^3^. However, their booking revenue has remained stagnant, and the proportion of guests opting for green hotels continues to be minimal^18^. Researchers have determined that there exists a fragile correlation between customers’ expressed favourable assessments and their patronage of green hotels^19,20^, and the reason for these visits is conspicuously under-researched^2^.

Understanding the fundamental elements influencing consumers’ intentions to engage in green hotel visits is crucial for hotels to optimise their operations and effectively meet guests’ environmental preferences^2^. However, rather than examining customers’ green purchase patterns concerning particular products or services, prior research in green marketing has concentrated on broad green purchase behaviour^11^. It may be difficult to extrapolate results from these studies to green hotel visits^12^. More importantly, a single theory is used in the majority of earlier studies that adopt a theoretical lens to analyse consumers’ green hotel visits^12^. Many other aspects that play a part are sometimes left out^21^. Hence, it is necessary to integrate theories and create a thorough integrated psychological research framework for studying green hotel visits^7,11^.

The theory of planned behaviour (TPB) is the most popular theoretical model for predicting consumers’ green hotel visits^9^. However, several objections were made regarding the validity and predictive power because the results of these investigations are sometimes contradictory^22^. For example, Haq, et al.^7^ reported that attitude cannot influence intention to visit green hotel, Niloy, et al.^1^ found that subjective norm insignificantly correlates with green purchase intention whereas Wang, et al.^12^ reported that perceived behavioural control cannot lead to intention to stay at green hotels. Besides, some studies have empirically confirmed that there is an interrelationship between constructs in certain situations^19,20,23^, although the current practices within TPB do not confirm any role of mediation^24^. The results of such studies have been in contrast to those of studies that rely on models that have their roots in the TPB, which demonstrates a lack of explanation of the relationships between the various constructs^12^.

Besides, TPB cannot sufficiently characterise the variability in behavioural intention^25^, such as green hotel visits^12^, since it is based on an expectancy-value theory of one’s behaviour based on self-interests^14^. Detailly, people’s assessments of or involvement in green hotel visits under TPB can reveal their perspectives, whether positive or negative^19^. However, consumers’ love of the environment or their emotional ties to the environment and future generations may have an impact on their opinions, whether favourable or unfavourable regarding staying at green hotels^26^. Subjective norm measures the degree to which an individual is impacted by their social environment and whether or not social pressure promotes staying at green hotels^20^. However, an individual’s internalised norm perception of the appropriateness of staying at green hotels can also influence the intention to visit^20^.

Moreover, researchers have shown that TPB has greater predictive power in green hotel visits when compared to value/norm orientation theories^27,28^ because its central component: perceived behavioural control reflects an individual’s opinion on how easy or difficult (i.e., time, resource, money, or opportunity) it is to perform a given behaviour^29^. However, the impact of barriers to visiting green hotels may be disregarded by consumers who have greater environmental values, beliefs, and norms about the preservation of the environment and natural resources^12^. To improve the explanatory power of behaviour in various contexts, the TPB needs to be expanded by adding new constructs^7,30,31^.

Value is seen as a trans-situational goal that varies in degree of relevance and serves as a guiding principle in one’s conduct^32^ because it is typically constant over an extended period^33^. Recent research revealed that people’s intentions to stay at green hotels are greatly influenced by their values, beliefs, and norms^15,26,34^. The value-belief-norm theory (VBN) is a suitable framework in environmental psychology because of its primary focus on values and personal moral norms based on altruistic environmental beliefs^21^. Several researchers showed that the VBN appears to complement the TPB for predicting consumers’ green hotel visits^12,21^.

However, compared to the TPB, the VBN was less effective in explaining high-cost environmental behaviours^27^. This is because (1) it is based on an individual’s capacity to act in an environmentally conscious manner without placing a premium on cost consideration (i.e., perceived behavioural control in TPB)^26^; (2) previous research did not differentiate between altruistic and biospheric values^33^; (3) the measurement of egoistic value is not valid for capturing the entirety of the population^28^ and (4) the VBN only considers an individual’s ecocentric attitude, which captures their concerns about society and its environmental protection-related issues, it ignores an individual’s positive or negative attitudes can be expressed by evaluating or engaging in specific real-world pro-environmental behaviours (i.e., implicit attitude in TPB) (e.g., attitudes towards visiting green hotels)^19^. Thus, although they are distinct theories, the TPB and VBN complement one another^21^. There seems to be a lack of application of the interplay between value, attitude, norm, and behaviour among the TPB and VBN in the literature on green hotel visits^26^.

Furthermore, the majority of research on green hotels has focused on Western countries (e.g., USA)^6^ and a small number of Asian countries or regions such as South Korea, Hong Kong, and Taiwan^2,10^, developing countries’ conditions have received less attention^20^. The fact that China’s tourism and hospitality sectors will account for around 3.96% of GDP in 2021 illustrates how these sectors support economic expansion^35^. Although the number of green hotels in China has increased significantly, some industry research reports and empirical investigations have shown that Chinese consumers are not very concerned about or knowledgeable about green hotels^22^. In comparison to Western nations, where the systematic framework is weak, research on green hotel stays is still in its early stages in China, and there is a dearth of literature to review^19^. More precisely, when it comes to green purchase attitude, knowledge, social norm, and intention, consumers in Eastern and Western countries have quite different collectivistic and individualistic values^36,37^. Some antecedents such as the egoistic value of green purchasing behaviour would not produce reliable results when they were designed and implemented in very collectivistic countries^12^.

In addition, Chinese citizens are generally regarded as some of the most environmentally conscientious individuals, which makes this particularly noteworthy^9^. There is an increasing demand for green consumerism as individuals become more environmentally and health-conscious^38^. For example, about 80% of Taiwanese visitors favour sustainable tourism and about 90% of travellers stated they would select at least one green hotel for their trip^9^. Chinese youth, in particular, are beginning to recognise the environmental advantages of green consumption^39^ and are more likely to embrace a sustainable lifestyle with heightened environmental consciousness^38^. This is important because young consumers are the future of travel, are more likely to purchase novel products and services advertised, and control a large portion of the market for different hotels^12^. More thorough research on Chinese youth’s green consumption is thus required^38^ as is an understanding of young people’s behavioural intentions to make green consumption, which has significant theoretical and practical implications^40^.

**Fig. 1:**
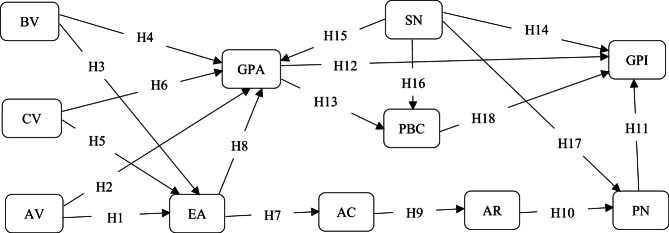
Theoretical framework of study.

Therefore, this study aims to contribute to the body of knowledge within the larger framework of the young green hotel visitor paradigm by evaluating the psychological components of the TPB and VBN (See Fig. 1). The main research objective is to investigate the interrelationship between TPB’s components, VBN’s components, and young consumers’ intentions to visit green hotels. By objectively examining the interrelationship of constructs among two theories, this study aims to present a novel integrated framework within the context of the investigation. This study will assess the effect of a multidimensional concept of value on implicit attitude and ecocentric attitude. This study will assess the effect of ecocentric attitude on implicit attitude and this study will also assess the interrelationship between subjective norm, implicit attitude, perceived behavioural control, personal norm, and intention. The results will contribute to the TPB and VBN by merging them to broaden the understanding of Chinese young people’s green hotel visits concerning upcoming green hotel operations and their possible benefits for achieving sustainable objectives.

## Literature review and hypotheses development

### Value-belief-norm theory

The VBN was created to comprehend people’s pro-environmental behaviour^34^ which derived from the moral norm-activation theory of altruism^41^. The moral norm-activation theory of altruism mostly concentrates on pro-environmental efforts in the private sector, ignoring non-activist eco-friendly activities in green marketing^26^. Thus, VBN by emerging the different types of pro-environmental values and ecocentric attitudes and taking into consideration human pro-environmental intention and behavioural values^42^. Accordingly, an individual’s values (altruistic, biospheric, and egoistic) affect their beliefs about their ecocentric attitude – which in turn affects their pro-environmental behaviour and how they behave in private, environmental-significant sectors or otherwise^43^.

Recent studies using VBN found that it could predict travellers’ intentions to stay at green hotels^14,38^. Nevertheless, some researchers argued that the VBN has less predictive power in high-cost pro-environmental behaviours compared with the TPB^18,26^. This is because behavioural costs and restrictions are not taken into account by the VBN^27^, which is a theory driven by personal values^44^. More importantly, because some civilisations have strong collectivistic value orientation^37^, the egoistic value may not be the appropriate antecedent to use when predicting consumers’ visits to green hotels across all settings since collectivistic value might lend credence to the claim that egoistic value and green hotels visits are related^18,28^.

#### Altruistic value

Altruistic value refers to individuals’ concerns for the well-being of others and society as a whole^27^. It represents the act of performing good behaviours^33^ without anticipating anything in return^45^. Altruistic value is seen as an environmental problem^46^ and a shared value when individuals act in an environmentally friendly manner^47^. An optimistic outlook on life is more likely among those who place a high value on altruism and who have a strong sense of connection to nature^47^. Perceptions of how willingly guests are to sacrifice comfort or luxury^47^ to conserve the environment are the main factors influencing hotel guests’ decisions to stay at green hotels^26^. Customers who choose green hotels over traditional ones are demonstrating their concern for the environment and the next generation^48^. This is also the main reason why the number of visitors staying at green hotels has been steadily increasing^10^.

Certain studies have shown how altruistic value influences one’s ecocentric attitude and green purchase attitude towards purchasing green products or services. For example, Eid, et al.^14^ found that altruistic value positively influences consumers’ ecocentric attitudes towards visiting green hotels in Egypt whereas Prakash, et al.^46^ found that altruistic value positively influences consumers’ ecocentric attitudes towards purchasing eco-friendly packaged products in India. In addition, altruistic value positively improves consumers’ attitudes towards patronising green hotels for the welfare of others in China^26^ while keeping positive attitudes in mind among the Philippines consumers^49^. Nevertheless, the literature on altruistic value and related attitudes towards green hotels is limited compared with other traditional psychological constructs^45^. Hence, this study proposes that:

##### H1

Altruistic value positively influences consumer ecocentric attitude.

##### H2

Altruistic value positively influences consumer implicit attitude.

#### Biospheric value

Individuals are more committed to the environment when they feel a psychological bond with and are familiar with nature^34^. Therefore, when people encounter environmental issues, the biospheric value may offer the most plausible explanation for certain pro-environmental behaviours^21^. According to biospheric value, those who follow these principles are more likely to be pro-environmentalists and to exhibit a stronger concern for plants, animals, and other natural resources^28^. Because people who care about the environment are more prepared to deal with hassles when attempting to live sustainably^15^.

Biospheric value is the most important value type for explaining consumers’ visits to green hotels^26^. The distinct person-environment link under psychological attachment and orientation to nature is where attitude and intention are drawn from in the literature on green hotels^44,45^. For instance, Wang, et al.^28^ reported that Chinese consumers’ biospheric values significantly influence their green purchase attitude towards visiting green hotels and in another study by Wang, et al.^18^, consumers’ biospheric values positively influence their both ecocentric attitude and green purchase attitude to visit green hotels in China. In addition, D’Souza, et al.^15^ found that Australian consumers with biospheric values are likely to stay in green hotels. However, most past studies on green marketing did not differentiate between biospheric value and altruistic value orientation^28^. For example, previous studies merged biospheric value with altruistic value for predicting consumers’ green purchase behaviours^10,50^ as they considered altruistic value including individuals concerning future generations, vulnerable communities, and the planet as a whole, resulting in little is known about how biospheric value affects green hotel visits^12^. Thus, this study proposes that:

##### H3

Biospheric value positively influences consumer ecocentric attitude.

##### H4

Biospheric value positively influences consumer implicit attitude.

#### Collectivistic value (egoistic value)

Egoistic value places importance on one’s interests within the society^42^ which originates from people who are worried about their health and well-being^51^. Previous studies have demonstrated that egoistic value is generally negatively correlated with consumers’ attitudes and behaviours^46,52^. Because individuals with egoistic values have a strong selfish and competitive orientation and are less likely to practice pro-environmental behaviours^12^ and are typically autonomous, self-centred, and motivated to maximise their interests through self-interested outcomes^44^. For example, Eid, et al.^14^ found that egoistic value negatively influences guests’ intentions to visit green hotels and Hwang, et al.^53^ demonstrated that egoistic value negatively influences consumers’ environmentally friendly drone food delivery services.

The findings on egoistic value have recently been criticised by scholars who contend that it is still unclear if egoistic value also applies to more collectivistic civilisations^27,33^. Studies conducted in certain collectivistic societies showed that consumers’ pro-environmental attitudes and behaviours were unaffected by egoistic values. For instance, Ray, et al.^52^ reported that egoistic value insignificantly affects consumers’ intentions to use green hotels in India. Also, some studies even reported that egoistic value has a positive influence on consumers’ green purchase behaviour as Hong, et al.^38^ reported that there is a positive correlation between egoistic value and ecocentric attitude towards green consumption among Chinese youth.

One significant explanation is that collectivistic and egoistic values are completely opposed^26^. Collectivists give priority to the needs and desires of the group or society ahead of those of any one person^54^. They tend to be more helpful, cooperative, and goal-oriented than people with individualistic values^26^. They are more concerned with social and local issues, their significant-close problems, and environmental issues^55^, resulting in forgoing their self-interests in favour of the greater good of society^56^. In contrast, people with self-interest value orientation from individualistic societies will typically weigh the costs and benefits of participating in the green movement^57^. Therefore, in some situations, people with collectivistic values are more likely to act in ways that benefit the environment than people with individualistic values in green marketing^16^. Because collectivistic people believe that preserving the environment is essential to the welfare of society as a whole, egoistic values should not be accepted in highly collectivistic societies^16,28^.

Pro-environmental products and services are usually purchased by consumers who desire to gain advantages for themselves, such as improved health and well-being^48^. Green hotels are thought to be healthier than regular hotels^10^ because they incorporate features like live plants to control the amount of oxygen in rooms^58^. Attitudes and behaviours in green hotel visits are positively correlated with collectivistic value^27,28^ and individuals’ eco-friendly behaviours can be incentivised by collectivistic value^59^. For example, Tamuliene, et al.^60^ reported that collectivistic value positively influences tourists’ peer influence and finally green stays. Saleem^16^ revealed that collectivistic value positively influences guests’ inconvenience attitude and importance attitude to revisit green hotels in Pakistan. In addition, Wang, et al.^28^ reported that collectivistic value positively influences Chinese consumers’ green purchase attitudes towards green hotel visits. Nevertheless, it is still unclear how hotel guests’ intentions to visit and their collectivistic values and pro-environmental behaviour relate to one another^16,18^. Thus, these hypotheses are assumed for this study as:

##### H5

Collectivistic value positively influences consumer ecocentric attitude.

##### H6

Collectivistic value positively influences consumer implicit attitude.

#### Ecocentric attitude

The key belief component in the VBN is the ecological worldview, which influences subsequent belief components that can be evaluated using the new environmental paradigm^42^. Traditionally, the ecological worldview has been operationalised as a one-dimensional construct^45^ to assess an individual’s increased public involvement with society or the environment and its issues^19^. Accordingly, an ecological worldview places greater emphasis on protecting nature for the benefit of humanity and accords its moral significance^47^ due to the belief that nature possesses inherent and fundamental values^45^. Therefore, an ecological worldview represents an individual’s ecocentric attitude to green marketing^26,47^.

With a higher ecocentric attitude, people will show a strong interest in political, social, and other environmental preservation-related concerns^61^, and offer a range of viable solutions^62^. Tourists with higher significant ecocentric attitudes are more likely to stay at green hotels than those with lower significant ecocentric attitudes^63^ because they are aware that tourism-related businesses including building, transport, and shopping significantly contribute to environmental deterioration^34^. Certain investigations have shown that ecocentric attitude has a considerable impact on tourists’ knowledge of the negative environmental effects of green hotels^21,26^. For example, Wang, et al.^12^ reported that ecocentric attitude positively correlates to consumers’ willingness to stay at green hotels. However, some studies reported that ecocentric attitude has no role in influencing tourists’ intention to visit green hotels^18,26^. Thus, we propose:

##### H7

Ecocentric attitude positively influences awareness of consequence.

Moreover, consumers tend to process information directly, but some of it also creates implicit attitudes through unintentional associations^61,64^. The validation of implicit attitudes’ ability to predict consumer purchase intention and behaviour joins the ecocentric attitude’s influence^65^. Ecocentric attitude suggests a rationalised relationship managed by a deliberate evaluation procedure^61^. As opposed to ecocentric attitude, which consumers can control directly because it is perceived as a direct statement of their purpose to act or judge, implicit attitude is a set of associations that come into being spontaneously^66^. Accordingly, ecocentric attitude will probably concur with the inherent attitude that ecological issues need to be addressed to preserve health and quality of life^67^. But more importantly, ecocentric attitude believes that even if these were not problems, nature is still vital to preserve because of its transcendental aspects^45^.

Therefore, it is crucial to remember that ecocentric attitude derives from a biospheric value orientation, one that underlines the intrinsic importance of the environment, whenever implicit attitude requires further explanation^26^. In other words, tourists with ecocentric attitudes will support public environmental policy-making more, but this will only have a small impact on the preservation of the natural environment^68^, and tourists with implicit attitudes are more likely to stay at green hotels while travelling to any destinations^47^. Thus, one must first understand the relationship between ecocentric attitudes and implicit attitudes in order to comprehend tourists’ attitudes towards staying at green hotels. Sarabia-Andreu and Sarabia-Sánchez^61^ noted a favourable association between ecocentric attitude and implicit attitude and agreed that people’s use of attitudes towards environmental concerns varies. Hence, we propose that:

##### H8

Ecocentric attitude positively influences implicit attitude.

#### Awareness of consequence, ascription of responsibility

Awareness of consequence refers to a person’s awareness of the negative effects of not acting pro-socially for others or other ideals^69^. With higher environmental awareness of consequence individuals are more willing to purchase green products or services and are willing to pay a premium for them^70^. People act altruistically in support of environmental issues because they believe certain situations offer threats to others^27^. Previous studies showed that consumers’ decisions to recycle and engage in behaviours that conserve water, energy, and local resources are most strongly influenced by their level of environmental awareness^71,72^. Indeed, the moral-activation theory of altruism and VBN presuppose that people who believe that certain circumstances pose threats to others will significantly influence them could avoid those adverse consequences because they ascribed responsibility to themselves^42^.

However, many studies have supported omitting the ascription of responsibility as an antecedent of personal norm^73^ because they believe that the ascription of responsibility and personal norm are thought to be closely related, and because its measurement indicators may also be related to personal norm^21^. Moreover, other studies have supported the direct impact of awareness of consequence on personal norm^14,74^. In contrast, some investigations continued to show that one’s pro-environmental intention is a function of personal norm generated by the problem awareness and ascription of responsibility connection^26,27^. Since problem awareness and ascription of responsibility are direct antecedents of personal norm, it is still unclear whether the ascription of responsibility is a sequential mediator framework or mediator model^69^. As such, we propose that:

##### H9

Awareness of consequence positively influences ascription of responsibility.

##### H10

Ascription of responsibility positively influences personal norm.

#### Personal norm

Personal norm refers to an individual’s value system, which may provide rise to feelings of moral duty that drive or abstain from a specific action^20^. It is the primary variable in the process of norm activation for a particular behaviour performed based on moral responsibility in a particular setting^69^. Personal norm is an internalised norm in green marketing^27^, which refers to an independent motivation to adhere to pro-environmental behavioural standards because an individual’s eco-friendly behaviour is a result of personal norm brought on by environmental problems concerns and an assigned responsibility relationship^43^. For example, consumers’ preference for staying at green hotels has likely developed into a moral requirement, and their behaviour has grown more customised depending on how they view the environmental aspects of the establishments^24^.

Previous studies revealed that while there is some resemblance between personal norm and subjective norm, there is also a very high correlation between personal norm and subjective norm in explaining intention under specific conditions^30^. However, since adding personal norm can significantly enhance some explained variance^20^, it should be considered an important predictor of an individual’s green purchase behaviour^14,43^. For example, personal norm is thought to be the important and main independent variable of intention to visit green hotels^20^ while Wang, et al.^27^ added personal norm in the TPB, which significantly improved forecasted consumers’ visits to green hotels. Therefore, we propose that:

##### H11

Personal norm positively influences intention.

### Theory of planned behaviour

The TPB is the most effective explanation for illuminating human pro-environmental behaviours^75^. Four elements make up the TPB: attitude, subjective norm, perceived behavioural control and intention^33^. Attitude is a person’s subjective evaluation of a particular behaviour, the subjective norm is their understanding of how others affect their decision-making, and perceived behavioural control is a person’s capacity and opportunity to engage in particular activities^30^. Concurrently, intention serves as a person’s mental motivation to place effort into performing a particular activity^30^. Intention is thought to be the best predictor of actual behaviour in marketing^76^ as it is for assessing the effectiveness of marketing strategies to foretell sales and market share^77^. As such, attitude, subjective norm, and perceived behavioural control come before intention.

The TPB has been widely used in green marketing to explain why consumers visit green hotels^7,78^. However, recent scholars have argued that it has less explanatory capacity when it comes to explaining consumers’ green purchase behaviours than value- or norm-orientation theories in some contexts^26,79^. Because the TPB is a behavioural theory that depends on a causal process^80^ that involves weighing people’s expected costs and benefits^18^ and rational consideration of people’s decision-making processes based on their self-interests^21^. Hence, it ignores other crucial factors including individual’s personal feelings, private norms, and decision-making criteria^18^. This supports Ajzen^30^ claims that if other constructs can account for a sizable amount of the variance in intention, they can be added to the TPB to predict consumers’ behavioural intention^78^.

#### Implicit attitude

Consumer’s implicit attitude refers to people’s attitudes that are favourable or unfavourable feelings towards a specific object and phenomenon that live outside of environmentally conscious awareness^66^. In other words, customers who have an implicit eco-friendly attitude will engage in particular environmentally responsible actions for the pure pleasure of doing so^81^. In the VBN, ecocentric attitude, in contrast, results from environmental deliberate reflection^82^, which adopts pro-environmental activities in order to avoid the unfavourable effects of doing otherwise^81^. Thus, consumers’ positive or negative ecocentric attitudes can be expressed by a love of nature or having an emotional fondness for nature^44^. In contrast, in the TPB, visiting green hotels can also be required by evaluating or partaking in actual green hotel visitations^19^. Accordingly, implicit attitude was only evaluated using the degree to which a given object (e.g., green hotel) was associated with a person’s favourable or unfavourable evaluations^19^. Thus, ecocentric attitude is mostly related to public pro-environmental behaviour, while implicit attitude is more correlated with specific pro-environmental behaviour such as visiting green hotels^68^.

Overall, a favourable implicit attitude towards visiting a particular hotel can be developed by providing various facilities, such as service quality, safety, security protocols, and brand reputation^83^. Certain studies have shown how the relationship between consumers’ implicit attitudes and intentions to visit green hotels. For example, Pan, et al.^9^ reported that implicit attitude positively influences Z-generation tourists’ green hotel visit intention. Ferreira, et al.^78^ found that implicit attitude is the most important psychological factor that influences consumers’ intentions to visit green hotels. However, Haq, et al.^7^ revealed that consumer’s implicit attitude toward green hotels cannot influence their intention to visit green hotels. Hence, we propose the following hypothesis for testing:

##### H12

Implicit attitude positively influences intention.

Meanwhile, because affective reactions and implicit attitudes in general shape people’s overall evaluations and feelings about their capacity to perform a specific behaviour^20^, positive implicit attitudes also can influence people’s belief that they can perform that behaviour^84^. In other words, individuals who have high perceived behavioural control or a high level of perceived self-efficacy can over the behaviour itself^85^ since they have a positive overall evaluation and feelings about a specific object^20^. Thus, a person’s implicit attitude has positive effects on perceived behavioural control and behavioural intention^86^.

Many TPB-related studies reported that implicit attitude is the most important predictor of consumers’ perceived behavioural control and intention^1,87^. For example, Lin, et al.^88^ indicated that implicit attitude positively influenced perceived behavioural control, subsequently affecting one’s responsible environmental behaviour, while Wang, et al.^20^ showed that implicit attitude positively influenced guests’ perceived behavioural control towards visiting green hotels. Additionally, Al-Gharibah and Mahfod^6^ indicated that implicit attitude positively influenced one’s intention to visit green hotels and Dwivedi, et al.^89^ observed similar findings in India. Thus, we propose that:

##### H13

Implicit attitude positively influences perceived behavioural control.

#### Subjective norm

The literature has begun to explore the significance of the role of subjective norm^20^ because (1) the literature as it stands now seems to downplay the impact of subjective norm in affecting consumer behaviour. Sometimes, studies that rely on models derived from the theoretical framework of TPB^76^ do show a significant causal path from subjective norm to implicit attitude and perceived behavioural control of intention^20,90^; (2) although subjective norm and personal norm are two different normative predictors that are utilised to explain one’s psychological process under particular circumstances^19^, a person’s own consideration usually outweighs the impact of perceived social pressure^30^.

Subjective norm refers to conscious social pressure to participate in or refrain from participating in a specific behaviour^91^. Specifically, close friends, family members, co-workers, colleagues, or business partners might provide insight into the relevance of the subjective norm^92^. Thus, the subjective norm is the moral commitments or feelings of individuals^93^, and it stands for norms and values that are somewhat influenced by certain referents^94^. Accordingly, subjective norm refers to the social dynamic in which individuals interact with others who exhibit similar features, and these referents have a big impact on people’s choices and actions^95^.

Previous studies have shown how subjective norm influences consumers’ green hotel visits. For example, Ferreira, et al.^78^ found that subjective norm directly influences consumers’ intention to visit green hotels. Tamuliene, et al.^60^ reported that subjective norm is an important predictor of tourists’ green hotel stays. However, certain studies reported that subjective norm has no role in influencing travellers’ intentions to visit green hotels, for example, Kumar and Mohan^96^ reported that subjective norm is only TPB’s components cannot influence consumers’ green purchase intention whereas Wang and Wong^97^ found that subjective norm insignificantly influences consumers’ green hotel selection. Hence, the following hypothesis is proposed:

##### H14

Subjective norm positively influences intention to visit green hotels.

Meanwhile, recent studies have demonstrated that people’s attitudes towards an object influence their ability to do a certain behaviour as well as their information of positive or negative attitudes towards it^19,92^. Subjective norm can be viewed as an indirect source of information that has a substantial impact on an individual’s perceptions and feelings of capacity to visit a destination^98^. Positive or negative opinions about green hotels received from significant others will influence an individual to possess a positive or negative attitude^19^, and an individual’s perceived ability to visit a destination is also heavily based on one’s initial knowledge and images of that place^99^. This is particularly true in highly collectivistic societies where individuals share similar values and beliefs and have emphasised the concept of the essential harmony of collectivism^20^. Thus, individuals are more likely to choose a product or service (e.g., green hotel) if they are aware of some information about it from their significant others^18^. Wang, et al.^20^ and Wang, et al.^18^ reported that subjective norm significantly influences consumers’ implicit attitudes and perceived behavioural control to patronise green hotels. Hence, the following hypotheses are proposed:

##### H15

Subjective norm positively influences implicit attitude.

##### H16

Subjective norm positively influences perceived behavioural control.

Furthermore, subjective norm is an external norm that represents a person’s value system that is impacted by social pressure^97^, whereas personal norm is an internalised norm that denotes an individual’s value system in a specific setting^19^. Thus, individuals can use subjective norm to determine whether a given behaviour option is advantageous or easy to perform, and whether or not significant others support it^100^. This implies that the opinions of significant others may have an impact on an individual’s personal perceptions, feelings, moral obligations, or accountability when engaging in such behaviours^27^. In other words, it is expected subjective norm will influence consumer’s personal norm in green marketing since an individual’s sense of obligation towards a particular pro-environmental behaviour (e.g., green hotel visit) can be activated by the perceived level of social pressure from their significant others^101^. Han, et al.^69^ reported that subjective norm is an important antecedent of personal norm towards eco-cruise activities while Liu, et al.^100^ found that subjective norm is an important predictor of personal norm towards green purchase intention. Thus, we propose that:

##### H17

Subjective norm positively influences personal norm.

#### Perceived behavioural control

Perceived behavioural control is defined as an individual’s perceived ease or difficulty in performing a certain activity^102^. This indicates that perceived behavioural control is influenced by control beliefs^86^. Perceived behavioural control is a measure of a person’s perceived level of comfort or confidence in conducting a specific behaviour^100^; on the other hand, it is a measure of a person’s perception of whether the behaviour is easier to perform than behaviours they perceive to be difficult and over which they have less control^92^. As a result, people choose to act in a particular way when the obstacles to doing so are manageable, such as when funds, time, abilities, opportunities, chance, and supporting factors are accessible^103^.

The more control an individual has over the factors keeping them from engaging in a behaviour, the more likely they are to do so^104^. In green marketing, perceived behavioural control is a crucial predictor of consumer pro-environmental behaviour^11,105^. For example, Pan, et al.^9^ found that perceived behavioural control significantly influences tourists’ green hotel visit intention whereas Ferreira, et al.^78^ also reported similar results. However, if behaviour is entirely volitional, perceived behavioural control will not influence intention^20,87^. Thus, we propose that:

##### H18

Perceived behavioural control positively influences intention.

## Methodology

### Data collection

In social science research, finding suitable samples to address questions of interest and obtaining an accurate sampling frame from both public and private institutions are challenging^106^. Hence, non-probability sampling is an alternative technique for choosing samples based on the researchers’ subjective assessments^107^. Purposive sampling was employed to choose respondents because it allowed researchers to choose the most qualified respondents using their expertise and professional judgement, thus, achieving research objectives^106^. According to Saunders, et al.^106^, purposive sampling is a technique where the researcher uses their judgement to choose each case that comprises the sample, with a special focus on choosing cases from a subgroup whose members are all similar. Hence, subjects are selected based on the study purpose with the expectation that each participant will provide unique and rich information of value to the study^108^. Many previous green hotel studies employed purposive sampling techniques for data collection and achieved the study’s objectives^2,34^.

The sample for this study was selected from university students because they are more knowledgeable about green consumption than aged consumers^3^ and more concerned about environmental issues than other age groups^109^. From hotel operators’ perspective, younger university generations will shape a different consumption pattern in the future^110^. For example, they dominated various hotel’s market share including traditional hotels and home-stay hotels, which may they have a higher willingness to purchase novel products or services in the future^12^. In addition, highly educated respondents will provide more accurate data than less educated respondents^111^. The questionnaires were distributed to university students who are pursuing their 3-year diploma, 4-year bachelor or master and above degree in Xuzhou City, Jiangsu Province, China. This is because Jiangsu Province had the third greatest population of undergraduate students, with Xuzhou accounting for about one-fifth of the total^33^.

The administered questionnaires were distributed to students of different majors who were present in class between 20 August to 20 September 2023 among six undergraduate universities in Xuzhou City. The network of contracts (assistant professors, lecturers, and associate professors) shares the questionnaire linkage with students in class. Students used WeChat or Alipay to scan the QR code and complete the questionnaire at www.wenjuan.com.Wenjuan.com is the largest free online survey platform with more than 10 million users in 2020, it focuses on providing questionnaire creation, distribution, management, collection and analysis services in China^23^. As compensation, each participant received a gift certificate (¥ 5, equal to USD 0.7).

Finally, 406 useable surveys were collected, surpassing Hair Jr, et al.^112^’s recommendation of a minimum sample size of 200 to offer an acceptable margin of error and between 10 and 20 cases per parameter for structural equation modelling^113^. This sample size also exceeds a minimum sample size of 384 is recommended when the target population is unknown or infinite based on Cochran’s Formula^114^. In addition, informed consent was obtained from all individual participants included in the study since written informed consent was obtained before the survey and the questionnaire and methodology for this study involving human participants were reviewed and approved by the Business School Research Ethics Review Committee of the Xuzhou University of Technology and all procedures performed were conducted by the relevant guidelines and regulations.

### Questionnaire operationlisation

The close-ended questionnaire with a five-point Likert scale to measure the constructs because (1) a five-point Likert scale will more likely produce higher mean scores and will make data comparison a much easier process compared with a ten-point Likert scale^115^; (2) the use of a five-point Likert scale rather than a seven-point Likert scale made it feasible to compare reliability coefficients with other investigations^116^; and (3) all measure items were adapted from earlier study using a five-point Likert scale. All items were developed from previous studies due to these constructs’ items have been tested by several empirical studies. The questionnaire was translated into Chinese and then back-translated to confirm the accuracy of the meaning of each item. Indeed, we performed wording modifications on all measurement items to fit the green hotels visit context. That is, the measures for constructs utilised in this study were all related to green hotel visits’ decision-making process and behaviour. To ensure the validity of the developed questionnaire items, a pilot test with 30 respondents was performed to prevent any problems that may affect the quality of the obtained data. Meanwhile, the questionnaire items were checked with three hospitality and tourism-related scholars to ensure the content validity.

The first section includes VBN components: six items belong to altruistic value, four items belong to biospheric value, and five items belong to collectivistic value were adopted from Wang, et al.^27^ and Wang, et al.^28^; five items belong to ecocentric attitude were adopted from Leonidou, et al.^68^ and Hwang, et al.^53^; four items belong to awareness of consequence and three items belong to ascription of responsibility were adopted from Han^117^ and Wang, et al.^27^; and five items belong to personal norm were adopted from Wang, et al.^27^. The second section includes TPB components: three items belonging to implicit attitude were adopted from Wang, et al.^27^; three items belonging to subjective norm were adopted from Dwivedi, et al.^89^; three items belonging to perceived behavioural control were adopted from Nimri, et al.^11^; and four items belong to intention were adopted from Sultana, et al.^2^. The last section includes demographic characteristics such as age, gender, educational background, and frequency of visiting hotels in the past year/three years.

### Common method bias

Considering the effects of common method bias, respondents were guaranteed confidentiality and privacy with personal information and answers, and their participant’s ability to withdraw at any time is permitted. Furthermore, Harman’s single factor test was used to detect whether common method bias does affect data. The results showed the first factor accounted for 45.689% which is below the threshold of 50%^118^. Besides, the full collinearity of constructs was computed to comment on common method bias and the VIF value for each construct should be less than 3.3^119^. Results showed that all VIFs are less than 3.3 (See Table 1), indicating that the model can be considered free of common method bias.

**Table 1:** Convergent validity of the measurement model.

Construct (Cronbach’s Alpha)	Items	Item loading	CR	AVE	VIF
Altruistic value	1. I have given directions to strangers. 2. I have given money or donated goods to a charity or group (delete). 3. I have given money to a stranger who needed it. 4. I have pointed out a clerk’s error (delete). 5. I have let a neighbour whom I did not know too well borrow an item of some value to me (delete). 6. I have offered my seat on a bus or train to a stranger who was standing.	0.749 0.680 0.819	0.794	0.564	1.575
Biospheric value	1. Respecting the earth. 2. Unity with nature. 3. Protecting the environment. 4. Preventing pollution.	0.927 0.786 0.916 0.825	0.923	0.750	1.961
Collectivistic value	1. Help others in a time of need (delete). 2. Maintain warm relationships with others. 3. To do well in life, the help of friends is crucial. 4. Be interdependently related to others. 5. Feel part of a large group of people (delete).	0.885 0.882 0.825	0.899	0.747	2.139
Ecocentric attitude	1. Major political change is necessary to protect the natural environment. 2. Anti-pollution laws should be enforced more strongly. 3. Major social changes are necessary to protect the natural environment. 4. Humans are severely abusing the environment. 5. The earth is like spaceship with limited rooms and resources.	0.906 0.907 0.881 0.901 0.876	0.968	0.911	2.338
Awareness of consequence	1. Hotel industry causes pollution, climate change, and exhaustion of natural resources. 2. Hotel industry generates the environmental impacts on the neighbouring areas and wider environment. 3. Hotel industry causes environmental deteriorations like waste from rooms, restaurants, and other facilities, excessive use of energy/water. 4. Green hotels practicing energy/water conservation, waste reduction, and diverse eco-friendly activities help to minimise the environmental degradations (reverse-coded) (delete).	0.925 0.900 0.914	0.937	0.833	1.277
Ascription of responsibility	1. I believe that every hotel guest is partly responsible for the environmental problems caused by the hotel industry. 2. I feel that every hotel guest is jointly responsible for environmental deteriorations caused by the hotel industry. 3. Every hotel guest must take responsibility for the environmental problems caused by hotels.	0.813 0.884 0.853	0.887	0.723	1.000
Personal norm	1. I feel staying at green hotels and using environmentally friendly products/services would make me a better person. 2. I feel staying at green hotels make me feel as a morally obliged person instead of conventional hotels. 3. I feel staying at green hotels saving environment should be the first priority for a person like me. 4. I feel staying at green hotels is a moral obligation regardless of what other people to do. 5. I feel staying at green hotels saving energy as much as possible is my personal obligation (delete).	0.900 0.887 0.853 0.774	0.915	0.731	1.629
Implicit attitude	For me, stay at a green hotel when travelling is: 1. Good. 2. Desirable. 3. Positive.	0.943 0.958 0.962	0.952	0.800	1.638
Subjective norm	1. People important to me, think that I should stay at green hotels. 2. Most people who are important to me would want me to choose green hotels when travelling. 3. People whose opinion I value would want to stay at green hotels.	0.961 0.956 0.896	0.956	0.880	2.271
Perceived behavioural control	1. I have resources, time, and opportunities to stay at a green hotel when travelling. 2. I am confident that if I want, I can stay at a green hotel when travelling. 3. Whether or not I stay at a green hotel when travelling is completely up to me.	0.727 0.855 0.771	0.828	0.618	2.072
Intention	1. I am willing to stay at a green hotel when travelling. 2. I plan to stay at a green hotel when travelling. 3. I will make an effort to stay at a green hotel when travelling. 4. I am willing to spend extra to stay at a green hotel when travelling.	0.908 0.907 0.885 0.901	0.945	0.810	-

## Analysis and findings

This study adopts the Statistical Package for the Social Sciences for descriptive analysis and internal reliability test. Moreover, covariance-based structural equation modelling was used to test the hypotheses developed in this study. According to Hair Jr, et al.^120^, covariance-based structural equation modelling considers the constructs as common factors that explain the covariation between its associated indicators. This approach is in line with reflective measurement’s philosophy, which views the indicators and their covariations as expressions of the underlying structures^120^. Hence, covariance-based structural equation modelling is a more direct and precise method to empirically measure theoretical concepts^120,121^. Because this study intends to investigate the impact of VBN and TPB components on consumer intention to visit green hotels based on the VBN and TPB model, covariance-based confirmatory factor analysis and structural equation modelling were employed.

### Demographic analysis

Table 2 shows about 60.3% of female and 39.7% of female respondents responded to the survey. The dominant age group was 20 years old (34%); the majority of respondents are 4-years bachelor’s degree students (97.3%) and in their junior careers (39.1%). Most of these respondents patronised hotels less one time in past year (29.1%) and visited hotels at least two times in past three years (24.4%).

**Table 2:** Demographic characteristics of sample (*N* = 406).

Items	Characteristics	Frequency	Percentage (%)
Gender	Male	161	39.7
	Female	245	60.3
Age	Below 18	6	1.5
	18	73	18.0
	19	80	19.7
	20	138	34.0
	21	77	19.0
	22	20	4.9
	23	7	1.7
	24	2	0.5
	Above 24	3	0.7
Education level	3-years diploma	3	0.7
	4-years bachelor	395	97.3
	Master and above	8	2.0
Seniority level	Fresh	86	21.2
	Sophomore	125	30.8
	Junior	159	39.1
	Senior	32	7.9
	Other	4	1.0
Visit hotels within 1 year	Less 1 time	118	29.1
	Once	80	19.7
	Two times	92	22.6
	3–4 times	75	18.5
	5–6 times	17	4.2
	7–10 times	4	1.0
	More than 10 times	20	4.9
Visit hotels within 3 years	Less 1 time	33	8.1
	Once	85	20.9
	Two times	99	24.4
	3–4 times	94	23.2
	5–6 times	32	7.9
	7–10 times	13	3.2
	More than 10 times	50	12.3

### Measurement model test

The rule of thumb for assessing the practical significance of a factor is that the factor loading must be higher than 0.5; ideally more than 0.7^112^. Thus, items of factor loadings of below 0.7 (i.e., AV2, AV4, AV5, CV1, CV5, AC4, PN5) were dropped (except AV2 = 0.68). To test internal reliability, Cronbach’s alpha value should be more than 0.7 is considered^112^. The convergent validity was assessed by considering the composite reliability (CR) should be higher than 0.7 and the average variance extracted (AVE) value should be higher than 0.5. For assessing discriminate validity, the AVE should be higher than the maximum shared squared variance (MSV) and the average shared square variance (ASV), meanwhile, the correlation between each construct should be less than 0.9 ^122^. Therefore, convergent validity (See Table 1) and discriminate validity (See Table 3) were established.

**Table 3:** Discriminate validity of the measurement model.

Construct	AVE	MSV	ASV	1	2	3	4	5	6	7	8	9	10	11
1. AV	0.564	0.480	0.237	***0.751***										
2. BV	0.750	0.441	0.220	0.502	***0.866***									
3. CV	0.747	0.480	0.314	0.693	0.664	***0.864***								
4. PN	0.731	0.717	0.396	0.565	0.498	0.691	***0.855***							
5. EA	0.911	0.593	0.315	0.394	0.522	0.565	0.579	***0.954***						
6. AC	0.833	0.493	0.234	0.477	0.306	0.497	0.660	0.344	***0.913***					
7. AR	0.723	0.717	0.308	0.524	0.391	0.564	0.847	0.423	0.702	***0.850***				
8. IA	0.800	0.764	0.377	0.459	0.592	0.579	0.629	0.770	0.401	0.481	***0.894***			
9. SN	0.880	0.588	0.279	0.342	0.214	0.307	0.561	0.531	0.449	0.494	0.603	***0.938***		
10. PBC	0.618	0.588	0.284	0.399	0.365	0.386	0.512	0.549	0.426	0.436	0.604	0.767	***0.786***	
11. Intention	0.810	0.764	0.400	0.419	0.456	0.530	0.677	0.769	0.430	0.530	0.874	0.737	0.716	***0.900***

altruistic value (AV). Biospheric value (BV). Collectivistic value (CV). Personal norm (PN). Ecocentric attitude (EA). Awareness of consequence (AC). Ascription of responsibility (AR). Implicit attitude (IA). Subjective norm (SN). Perceived behavioural control (PBC).

In addition, to assess the overall goodness of fit indices of the measurement model and structural model, certain model fit indices thresholds should be considered such as CMIN/DF below 3 is good and below 5 is permissible^112^. SRMR should be below 0.1, CFI should be more than 0.9, and TLI should be more than 0.9^123^. IFI should be more than 0.9, PCFI should be more than 0.5^122^, NFI should be more than 0.8, PGFI and PNFI should be more than 0.5, and RMSEA should be less than 0.1^124^. The results showed that CMIN = 1513.266, DF = 603, *p* < 0.05, CMIN/DF = 2.51 < 5, SRMR = 0.048 < 0.1, CFI = 0.945 > 0.9, NFI = 0.912 > 0.8, IFI = 0.945 > 0.9, TLI = 0.935 > 0.9, PGFI = 0.676 > 0.5, PNFI = 0.782 > 0.5, PCFI = 0.81 > 0.5, RMSEA = 0.061 < 0.1, indicating an acceptable good measurement model.

### Structural model test

**Fig. 2:**
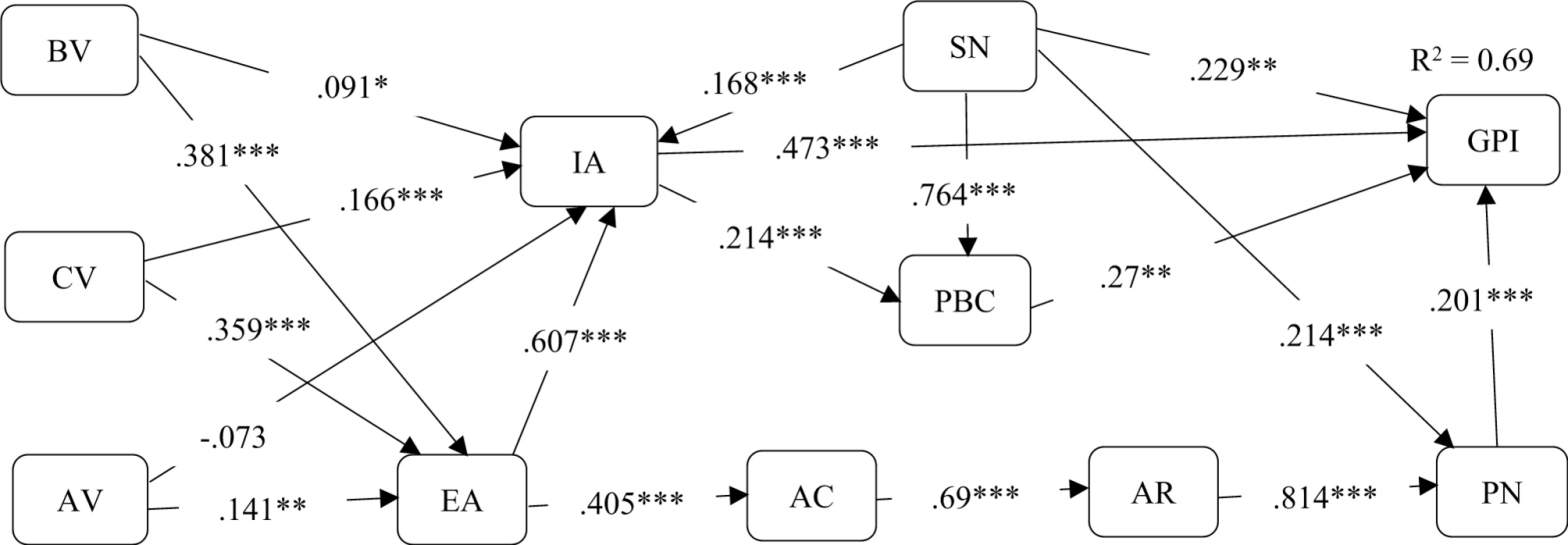
Outcomes of the study. * denotes *p* < 0.05, ** denotes *p* < 0.01, *** denotes *p* < 0.001. altruistic value (AV). Biospheric value (BV). Collectivistic value (CV). Personal norm (PN). Ecocentric attitude (EA). Awareness of consequence (AC). Ascription of responsibility (AR). Implicit attitude (IA). Subjective norm (SN). Perceived behavioural control (PBC).

The model fit indices of the structural model showed CMIN = 2491.67, DF = 640, *p* < 0.05, CMIN/DF = 3.893 < 5, CFI = 0.888, NFI = 0.854 > 0.8, IFI = 0.889, TLI = 0.877, PGFI = 0.649 > 0.5, PNFI = 0.778 > 0.5, PCFI = 0.808 > 0.5, RMSEA = 0.085 < 0.1. Ho^125^ suggested that there were at least three indices to be met to make the model fit. Thus, the overall model fit indices demonstrated an acceptable model fit and the outcomes are illustrated in Fig. 2; Table 4 accordingly.

**Table 4:** Results of the study.

Hypothesis	Parameter	Estimate	*p*-value	C.*R*.	Decision
H1	Altruistic value→ecocentric attitude	0.141	0.006	2.767	Supported
H2	Altruistic value→implicit attitude	-0.073	0.08	-1.75	Rejected
H3	Biospheric value→ecocentric attitude	0.381	***	7.693	Supported
H4	Biospheric value→implicit attitude	0.091	0.031	2.158	Supported
H5	Collectivistic value→ecocentric attitude	0.359	***	7.270	Supported
H6	Collectivistic value→implicit attitude	0.166	***	3.871	Supported
H7	Ecocentric attitude→awareness of consequence	0.405	***	7.897	Supported
H8	Ecocentric attitude→implicit attitude	0.607	***	11.888	Supported
H9	Awareness of consequence→ascription of responsibility	0.690	***	14.954	Supported
H10	Ascription of responsibility→personal norm	0.814	***	17.753	Supported
H11	Personal norm→intention	0.201	***	5.679	Supported
H12	Implicit attitude→intention	0.473	***	11.506	Supported
H13	Implicit attitude → perceived behavioural control	0.214	***	5.079	Supported
H14	Subjective norm → intention	0.229	0.002	3.043	Supported
H15	Subjective norm→implicit attitude	0.168	***	4.504	Supported
H16	Subjective norm→perceived behavioural control	0.764	***	16.328	Supported
H17	Subjective norm→personal norm	0.214	***	6.371	Supported
H18	Perceived behavioural control→intention	0.270	0.002	3.096	Supported

The results of this study showed that altruistic value positively influences ecocentric attitude since β = 0.141, *p* < 0.05 and insignificantly influences implicit attitude as β = -0.073, *p* > 0.05, hence, H1 is supported but H2 is rejected. Results showed that biospheric value positively influences ecocentric attitude (β = 0.381, *p* < 0.05) and implicit attitude (β = 0.091, *p* < 0.05), thus, H3 and H4 are supported. Collectivistic value positively influences ecocentric attitude since β = 0.359, *p* < 0.05 and implicit attitude (β = 0.166, *p* < 0.05), thus, H5 and H6 are supported. Our results showed that ecocentric attitude positively influences awareness of consequence (β = 0.405, *p* < 0.05) and implicit attitude (β = 0.607, *p* < 0.05) respectively, hence, H7 and H8 are supported. There is a positive correlation between awareness of consequence and ascription of responsibility (β = 0.69, *p* < 0.05), thus, H9 is supported. Further, the ascription of responsibility positively influences personal norm as β = 0.814, *p* < 0.05, thus, H10 is supported. Results showed that personal norm positively influences intention since β = 0.201, *p* < 0.05, thus, H11 is supported.

For TPB-related constructs, the results of this study showed that implicit attitude positively influences intention as β = 0.473, *p* < 0.05, thus, H12 is supported. Meanwhile, there is a positive correlation between implicit attitude and perceived behavioural control since β = 0.214, *p* < 0.05, thus, H13 is supported. Subjective norm had a positive influence on intention (β = 0.229, *p* < 0.05), implicit attitude (β = 0.168, *p* < 0.05), perceived behavioural control (β = 0.764, *p* < 0.05), and personal norm (β = 0.214, *p* < 0.05) respectively. Hence, H14, H15, H16, and H17 are supported. In addition, perceived behavioural control positively influences intention to visit green hotels since β = 0.27, *p* < 0.05, thus, H18 is also supported.

## Discussion

Researchers should carefully evaluate the relationship between individuals’ values, attitudes, norms, and intentions to patronise green hotels^27^. Previous studies showed that altruistic value, biospheric value, and collectivistic value positively influence consumers’ ecocentric attitudes and, subsequently intention to make pro-environmental behaviours^21,28^. Our results showed that altruistic value, biospheric value, and collectivistic value positively influence consumers’ ecocentric attitudes towards visiting green hotels. This means that consumers who care about other people’s well-being, the environment and natural issues, and group-oriented goals are more likely to have a favourable attitude towards maintaining and protecting the environment and natural resources through visiting green hotels.

Previous studies found that collectivistic value and biospheric value positively influence consumers’ implicit attitudes towards a particular object^33,126^. The results of this study showed that biospheric value and collectivistic value positively influence one’s implicit attitude towards visiting green hotels. This means that people who value the environment and are cooperative are more likely to favourably assess the qualities of green hotels. Generally, they have positive overall evaluations of green hotels’ products and services. Certain studies found that altruistic value positively influences consumers’ implicit attitude and intention to visit green hotels^10,26^. However, our results showed that there is an insignificant negative correlation between altruistic value and implicit attitude. This indicates that people who prioritise the welfare of others did not have an impact on how green hotels were evaluated compared with those concerned about the environment and natural resources and placing group goals such as protecting the environment is beneficial for the next generation as a priority.

Previous studies found that individuals’ ecocentric attitudes positively influence their environmental awareness of the consequences of non-environmental purchase behaviours, which in turn lead to their attribution of responsibility to avoid such actions, then influence their personal judgement of engaging in such behaviours, and ultimately influence their intentions to engage in pro-environmental behaviours^26,27,42^. Results of this study showed that ecocentric attitude positively influences awareness of consequence, then ascription of responsibility, further personal norm, and lastly their intentions to visit green hotels. This means that consumers who are concerned about environmental and natural resources issues affect their consequence consideration of taking non-pro-environmental behaviours, they will attribute negative living environmental conditions such as global warming, and haze to their responsibility of not taking pro-environmental behaviours. Then, they will feel performing a specific green purchase behaviour such as visiting green hotels will make them better people and feel a positive moral obligation regardless of what other people do, which ultimately positively influences their green hotel visits.

Some scholars suggested that ecocentric attitudes may boost implicit attitudes in performing pro-environmental behaviour^61^. Our findings demonstrated that consumers’ ecocentric attitudes positively influence their implicit attitudes towards visiting green hotels. This suggests that people’s specific attitudes on evaluating green hotels are substantially influenced by their environmental attitudes towards the general public, protecting the environment and natural resources.

Furthermore, many TPB’s related studies reported that implicit attitude, subjective norm, and perceived behavioural control are important antecedents of green purchase behaviour^1,127^. Results of this study showed that implicit attitude, subjective norm, and perceived behavioural control all positively influence intentions to visit green hotels. This means that consumers’ positive evaluations of green hotels’ attributions and functions, perceived social pressure from their significant others e.g., close friends, relatives, colleagues, parents, and self-confidence beliefs e.g., time, resources, and money positively influenced their intention to select green hotels.

Some studies revealed that implicit attitude positively influences consumers’ perceived behavioural control to conduct a particular pro-environmental behaviour^20,88^. Our results found that there is a positive correlation between implicit attitude and perceived behavioural control. This suggests that a person’s perception of their ability to stay at green hotels was significantly influenced by their overall assessment of the green features of green hotels.

In addition, certain studies reported that subjective norm has a beneficial impact on implicit attitude, perceived behavioural control, and personal norm in engaging in pro-environmental behaviour^19,97^. Results of this study showed that subjective norm positively influences implicit attitude, perceived behavioural control, and personal norm respectively towards visiting green hotels. This means that individuals’ significant others’ opinions such as close friends, relatives, co-workers, classmates, and neighbours have a significant impact on their positive or negative evaluations of green hotel attributions, perceived abilities and confidence to visit, and personal feelings and moral obligations to visit green hotels.

### Theoretical contributions

Certain theoretical implications can be concluded from this study. The nature of the interaction between values, attitudes, and behaviours is complex^26^, and value is seen to be one of the key antecedents of eco-friendly activity^32^. First, the majority of previous studies have centred on the apparent impact of altruistic value appeals in marketing campaigns, yet a necessity arises to delve further into the understanding of the effects of altruistic value on consumers’ decisions in green marketing^50^, especially compared with other traditional psychological constructs^26^. The current study found that altruistic value is an important predictor of ecocentric attitude, however, it insignificantly influences implicit attitude towards visiting green hotels.

Second, many previous studies merged biospheric value with altruistic value as one construct for predicting consumers’ pro-environmental behaviours^10,43^. This implies that previous studies did not distinguish the role of biospheric value and altruistic value in influencing consumers’ green purchase behaviours, resulting in confusing results^33^. The results of this study showed that biospheric value is an important predictor of implicit attitude as well as it has the largest influence on consumers’ ecocentric attitude compared with collectivistic value and altruistic value towards visiting green hotels.

Third, using egoistic value to predict consumer green purchase behaviour is not acceptable in all circumstances, especially in highly collectivistic value-orientation societies^28^. Hence, some scholars suggested that researchers should adopt collectivistic value instead of egoistic value in predicting consumers’ pro-environmental behaviours in such societies^18,28,97^. This study found that collectivistic value favourably influences individuals’ ecocentric attitude and implicit attitude towards safeguarding environmental resources and visiting green hotels. Thus, future studies should consider the influence of collectivistic value in influencing one’s pro-environmental behaviour in highly collectivistic value orientation societies.

Furthermore, consumers’ positive or negative evaluations of green hotel qualities can influence their willingness to frequent green hotels^76^, but their ecocentric attitudes towards environmental issues and concerns can also influence their intention to patronise green hotels^20^. However, few research has examined the impact of both features of ecocentric attitude and implicit attitude on green hotel visits^19^, and even fewer have investigated the interaction between ecocentric attitude and implicit attitude in green marketing^47^. Our findings indicate that ecocentric attitude and implicit attitude should be considered as predictors of green hotel visits. Meanwhile, our results showed that there is a clear relationship between ecocentric attitude and implicit attitude. Future research should separate the influence of ecocentric attitude and implicit attitude on green hotel visitation and explore the interaction between ecocentric attitude and implicit attitude when predicting consumers’ intention to visit green hotels.

Fifth, the VBN proposed that there is a clear causal relationship between the components of beliefs (i.e., ecocentric attitude -> awareness of consequence -> ascription of responsibility -> personal norm)^41^. However, many prior studies did not use entire belief components based on their objectives for predicting consumer pro-environmental behaviour^21,34^. For example, Harland, et al.^128^ demonstrated that the ascription of responsibility is closely related to personal norm and its measurement indicators may be similarly related to personal norm, too, possibly causing the issues of reliability or validity in data analysis. Hence, some researchers have supported excluding the ascription of responsibility as a construct in the VBN^21,73^. According to this study, there is a direct, statistically significant association between ecocentric attitude, awareness of consequence, ascription of responsibility, personal norm, and intention to stay at green hotels. This provides a more comprehensive causal explanation of how consumers’ pro-environmental values translate into beliefs, subsequently on personal sense of obligations, and ultimately on green hotel visit intentions. This study recommends that future research use entire belief components to predict consumers’ intention to stay at green hotels because the results can provide a more thorough knowledge of consumer behaviour.

Sixth, the TPB suggested subjective norm is a significant predictor of consumers’ intention while the VBN proposed personal norm being a significant antecedent of consumers’ green purchase intention. However, researchers should investigate the potential impact of subjective norm on personal norm^20^ as well as the subjective norm and personal norm as parallel antecedents of consumers’ visits to green hotels^19^. Our findings showed that subjective norm and personal norm significantly influenced one’s intention to stay at green hotels. Meanwhile, the results of this study found that subjective norm also significantly influenced personal norm. Therefore, in order to increase the predictive power of consumer green hotel visitation, future studies should include both subjective norm and personal norm as explanatory normative elements.

Seventh, subjective norm is the most complicated predictor for explaining consumer’s green purchase behaviour^104^. This is because some studies’ results showed that subjective norm ineffectively influenced consumers’ green purchase behaviours^1^ and other empirical studies showed that subjective norm had a direct and significant influence on implicit attitude, perceived behavioural control, and personal norm^92,97,100^. Our results revealed that subjective norm significantly influenced consumers’ implicit attitude, perceived behavioural control and personal norm, and thus their intention to patronise green hotels. Thus, future studies should explore how the subjective norm needs to be considered as an antecedent of other psychological constructs and intentions in green marketing literature.

Eighth, some studies revealed that perceived behavioural control cannot enable one to achieve their green purchase behaviour^14^. However, the basic explanation for why TPB appeared to have more explanatory power in situational characterised by high-cost behaviours when compared to normative orientation theories (e.g., VBN) is provided by the influence of perceived behavioural control^20^, which represents the level of confidence of an individual in performing the behaviour^129^. The results of this study showed that perceived behavioural control significantly influences consumers’ green purchase intention to visit green hotels. Thus, future research is necessary to take into account perceived behavioural control’s impact on consumers’ intentions to stay at green hotels.

In addition, a single theory is insufficient to explain one’s green purchase behaviour^130^. Despite being different theories, the TPB and VBN can be used in conjunction to predict consumers’ green purchase behaviour^21^. This study explored the possible relationship between different constructs of the TPB and VBN. Our findings demonstrated that biospheric value and collectivistic value had an impact on individuals’ ecocentric attitude and implicit attitude respectively. Altruistic value also significantly influenced ecocentric attitude. Additionally, there is a strong association between subjective norm and personal norm as well as a strong correlation between ecocentric attitude and implicit attitude. Therefore, future research can take into account combining both TPB and VBN to predict consumers’ intention to stay at green hotels due to the current merged research framework could provide more comprehensive explanations of the interrelationships of constructs on how consumers’ pro-environmental values influence their beliefs components, subsequently normative component and finally intentions to visit green hotels.

Last but not least, there has not been much research on green hotels in emerging nations like China^131^ with highly collectivistic cultures^37^. Sometimes, applying a theory or research framework in study for investigating a specific phenomenon in various regions or different cultural backgrounds will carry out different results. For example, some researchers argued that applying egoistic value as an important value predictor of consumers’ pro-environmental behaviour may not be suitable for highly collectivistic value orientation societies^12,18^. Chinese people are found to consider themselves more strongly as part of a larger whole and often prioritise the group’s needs over the individual’s needs than those in individualistic countries^37^. Therefore, the findings of this study offer a fundamental knowledge and research framework on how consumers’ intentions to stay at green hotels in a developing and highly collectivistic value orientation country based on the TPB and VBN.

### Practical implications

The results of this study have several useful applications. First, our results revealed that altruistic value has a positive impact on consumers’ ecocentric attitude. Green hotel operators should emphasise how adopting green operating strategies and practices benefits others, particularly regarding their environmental concerns. For example, green hotels should highlight that saving water and energy and reducing emissions will benefit customers families’ members, relatives, and the next generations. As a result, people with strong altruistic value orientation are more inclined to preserve the environment and natural resources, finally influencing their intentions to visit green hotels.

Second, our results demonstrated that biospheric value has a favourable impact on consumers’ ecocentric attitude and implicit attitude regarding visiting green hotels. Indicators of how much natural resources have been saved by green hotels should be provided. For example, green hotels should highlight that how many tons of water and how many energies they saved through implementing green practices. Customers would therefore believe that their green hotel patronage actions are actually preventing pollution.

In this study, collectivistic value has a beneficial influence on consumers’ ecocentric attitude and implicit attitude towards green hotel visits. Green hotel operators need to demonstrate that applying green techniques is not only helpful to themselves, the environment, and natural resources, but also aligned with the goals of the groups, communities, or countries. Because the majority of customers, residents, and countries are currently pursuing a human-nature balanced environmental atmosphere.

In this study, ecocentric attitude has a positive effect on implicit attitude and awareness of consequence. The primary goal of a green hotel is to show the differences between the qualities of green hotels and traditional/conventional hotels. Green hotels should publicise how other traditional hotels and their consumers have abused natural resources; this is why many hotel operators see green hotels as an alternative marketing technique to attract potential consumers.

Consumers’ awareness of consequence has a favourable influence on one’s ascription of responsibility. Therefore, green hotel operators should emphasise what green products (e.g., organic foods, recyclable products) or services (e.g., solar energy facilities) they can provide for potential consumers so that they understand that not adopting such products or services will have a negative impact on the environment and natural resources.

Because our findings indicated that ascription of responsibility has a significant effect on personal norm. Green hotels can promote the number of natural resources that can be conserved by everyone’s patronage behaviour. For example, green hotels’ guests can use some recyclable products or services (e.g., recyclable tissue, organic foods) to reduce negative impact on the environment. Such information may raise consumer perceptions of the obligation to adopt environmentally friendly behaviours by utilising green products or services offered by green hotels.

Our findings indicated that personal norm has a favourable impact on their decision to stay at green hotels. Green hotels can use information posted in their establishments (e.g., green information in the bulletin) to remind guests why they should conserve natural resources and how their actions can protect the environment. This will increase their consumers’ moral obligations to be pro-environmentalists.

Furthermore, this study showed that subjective norm has a positive impact on implicit attitude, perceived behavioural control, and personal norm towards visiting green hotels. Advertising is the most vital marketing strategy that green hotels should use. Green hotels should make it obvious what makes them environmentally friendly and how they can manage their limited natural resources to offer the same level of amenities as standard hotels. This will help them gain more positive public perception, thus influencing others to influence potential consumers to visit green hotels in the future.

Consumer’s intention to stay at green hotels was positively influenced by implicit attitude. Green hotels operators should emphasise that choosing to stay in one is preferable to staying in a traditional hotel. Green hotel should specifically state that they do not lower the quality of their products and services; rather, they provide their guests with high-quality or more green and nutritious products and services. Hence, potential guests will have a favourable attitude regarding visiting green hotels due to they have a positive overall evaluation of the attributes and functions of green hotels.

Last, perceived behavioural control has a positive effect on consumers’ intentions to stay at green hotels. Green hotel operators need to make sense for consumers regarding whether they have the time, opportunity, money, and resources to book and visit green hotels. This is because consumers typically believe that green hotels would sacrifice the quality of their products or services, charge more, and offer fewer options when vacationing. Therefore, to reduce consumers’ barriers to visiting green hotels, operators of green hotels should offer more information and facilities such as shuttle buses, booking information, and transparent prices for potential consumers.

### Limitations and future suggestions

This study had certain limitations. First, this study attempts to provide comprehensive explanations of how various values, beliefs, and norms influence consumers’ intentions to visit green hotels based on the TPB and VBN. There still many other theories are popularised in green hotel literature such as goal-framing theory or goal-directed model may be considered as complements to the TPB or VBN. Therefore, future studies may examine the promising correlations between different constructs across different theories. Second, this study was only conducted in China, particularly the Xuzhou City. Thus, the context will only apply to this area. It may vary across other regions or countries due to cultural differences and many other factors. Thus, the research model used in this study should be replicated and tested in other regions and countries to further confirm its usefulness and reliability.

Third, Jiangsu Province had the third greatest population of undergraduate students, with Xuzhou accounting for about one-fifth of the total^33^. However, the recognition and perceptions of green hotels and the ability to visit green hotels of students may vary due to economic imbalance in China and other factors. Future studies may consider using the probability sampling technique to select samples among all university students, the results of findings have more representativeness. Indeed, students have attitudes and knowledge most similar to the general public^132^. However, selecting university students as samples means that the rest of the population is ignored. For example, Han, et al.^133^ stated that older consumers are more willing to visit green hotels than younger people. Hence, the research respondents cannot represent the entire population or other demographic groups. Meanwhile, scholars demonstrated that university students are willing to spend their disposable income^134^ which dominates in the hotel’s marketing share^12^. Nevertheless, some researchers argued that students’ financial capacity and expenditure are considerably lower when compared to the working population^12^ and some scholars argued that highly income-level consumers have more pro-environmental attitudes and behaviours compared with lower income level consumers^135,136^. Hence, this study relies on students as a sample may weaken the study’s credibility and future studies should explore consumers’ green hotel visits based on various samples in different regions and countries.

Fourth, monetary incentives for data collection tend to show that they can affect representativeness, especially prepaid token incentives may result in a less representative sample composition than no incentives^137^. Thus, future studies may investigate consumers’ green hotel visits without incentives in surveys although this will increase the challenge in the data collection process. Furthermore, intention is the most robust predictor of an individual’s actual behaviour in marketing literature^30^. In certain circumstances, however, an individual’s actual behaviour is not always equivalent to one’s behavioural intention^138^. Future studies may delve into investigating students’ or consumers’ actual green hotel visitations based on this study’s research framework. In addition, recently, some scholars suggested that hedonic value should be considered as an additional value that appears particularly relevant to understanding people’s engagement in pro-environmental behaviours^57^ and some empirical studies confirmed its predictive capacity in green hotel marketing^27,139^. Many studies have shown that altruistic and biospheric values are generally prioritised over egoistic and hedonic values^57^. Hence, the current research considers retaining the original three types of pro-environmental values for predicting consumers’ green hotel visits. Future studies may consider merging four types of values in understanding consumers’ green hotel visits which enrich the green hotels literature.

Last, the findings of this study may provide a basic understanding of university students’ propensities towards visiting green hotels and thus enrich the green hotel literature in a specific developing country with a highly collectivistic value orientation. However, the results only play a foundational role in explaining Chinese university students’ intentions to visit green hotels. Henry^140^ recommends the replacement of student samples with a perfectly representative sample, instead recommending the replication of relationships on a differently representative sample. Thus, future studies should therefore be done to obtain the aforementioned restrictions, such as expanding the recruited respondents and testing them in different regions with different cultural backgrounds towards different types of green hotel practices to ensure the research framework’s utility and validity.

### Conclusion

Although there is much research on consumer intention to visit green hotels based on either the TPB or VBN, the decision-making process of visiting green hotels has not been thoroughly covered empirically in the green hotel literature. The current study explored how the TPB and VBN affect consumers’ intentions to visit green hotels. Altruistic value, biospheric value and collectivistic value have been proven to be significant predictors of ecocentric attitude. Meanwhile, biospheric value and collectivistic value were found to be reliable predictors of implicit attitude. The results found that there is a significant positive correlation between ecocentric attitude, awareness of consequence, ascription of responsibility, personal norm, and intention. Implicit attitude was found to be the most strongly influenced intention, followed by perceived behavioural control and subjective norm. Moreover, the results showed that subjective norm significantly influenced implicit attitude, perceived behavioural control, and personal norm towards intentions to visit green hotels. This study is one of the few studies that examined the interrelationships among TPB and VBN’s components that influence consumers’ green hotel visits in a developing country with a highly collectivistic value orientation. Therefore, this study will contribute to a better understanding of consumers’ green hotel visit intentions based on a combined theoretical framework, which contributes to the growth of the green hotel industry.

## Electronic supplementary material

Below is the link to the electronic supplementary material.

Supplementary Material 1

## Data Availability

The raw data supporting the conclusions of this study will be made available by the authors, without undue reservation.
