# Supplementary material for: The efficacy of the theory of planned behaviour and value-belief-norm theory for predicting young Chinese intention to choose green hotels
Source: Sci Rep. 2025 Apr 24;15:14332. doi: 10.1038/s41598-025-99447-1 (PMC12022360; doi:10.1038/s41598-025-99447-1)
Supplement: Supplementary file 1 — Supplementary Material 1 [file 41598_2025_99447_MOESM1_ESM.pdf]

EA1	EA2	EA3	PBC1	PBC2	PBC3	SN1	SN2	SN3
5	5	5	5	5	5	5	5	5
4	4	4	4	4	4	3	3	4
5	5	5	3	3	2	1	1	2
5	5	5	3	3	4	3	3	3
4	4	4	5	5	5	4	4	3
5	5	5	3	1	1	2	1	5
5	5	5	5	5	5	5	5	5
5	5	5	5	5	1	4	3	4
5	5	5	3	4	4	3	3	3
5	5	5	5	5	5	5	5	5
3	3	3	3	3	3	3	3	3
4	4	4	4	4	4	4	4	4
5	5	5	5	5	5	5	5	5
5	5	5	5	5	4	4	4	3
5	5	5	5	5	5	5	5	5
5	3	5	5	5	4	3	3	3
5	5	5	5	5	5	5	5	5
4	4	4	5	5	5	5	5	5
4	4	4	4	4	4	4	4	4
5	5	5	5	5	5	5	5	5
3	3	3	5	5	1	4	3	4
5	5	5	5	5	5	5	5	5
5	5	5	5	5	5	5	5	5
5	5	5	5	5	5	3	3	3
4	5	5	5	5	3	2	2	2
5	4	3	3	3	3	3	3	3
5	5	5	5	5	5	5	5	5
3	3	3	3	3	3	3	3	3
5	5	5	5	5	5	5	5	5
5	5	5	4	4	4	4	4	4
5	5	5	3	4	4	3	4	3
4	4	4	3	3	2	3	3	3
5	5	5	5	5	5	5	5	5
5	5	5	5	5	5	5	5	5
5	5	5	3	4	4	4	4	4
4	4	4	4	4	4	4	4	4
3	3	3	5	5	3	3	3	3
4	4	4	5	5	3	3	3	5
5	5	5	5	5	5	5	5	5
5	5	5	5	5	5	5	5	5
5	5	5	5	5	5	5	5	5
4	3	2	2	2	2	1	1	2
4	3	3	4	4	4	5	5	5
5	5	5	5	5	5	5	5	5
5	5	5	5	5	5	5	5	5
5	5	5	5	5	5	5	5	5
5	5	5	3	3	5	1	1	3
3	4	5	5	3	3	3	3	3
5	5	5	5	5	3	1	1	1
5	5	5	5	5	5	5	5	5
5	5	5	5	5	3	3	3	3
5	5	5	5	5	5	5	5	5
5	5	5	5	5	5	5	5	5

5	5	5	5	5	3	4	4	4
5	5	5	5	5	5	1	3	3
4	4	2	2	4	4	4	4	4
4	4	4	5	4	4	3	3	3
5	5	5	5	5	5	5	5	5
4	4	4	5	3	4	4	4	4
5	5	5	3	3	3	4	4	4
5	5	5	5	5	3	1	1	1
5	5	5	5	5	5	5	5	5
5	5	5	5	5	3	4	5	4
3	3	3	4	3	3	3	3	3
4	4	4	3	3	3	4	4	4
5	5	5	5	5	5	5	5	5
5	5	5	5	5	3	5	5	5
5	5	5	5	5	5	5	5	5
5	5	5	5	5	5	3	3	4
5	5	5	5	5	5	5	5	5
5	5	5	5	5	5	5	5	5
5	5	5	5	5	5	5	5	5
5	5	5	5	5	5	5	5	5
5	5	5	4	5	3	2	2	4
5	5	5	5	5	4	5	5	4
5	5	5	5	4	5	5	5	5
5	5	5	3	3	3	2	4	3
3	3	3	5	5	4	2	3	3
5	5	5	5	4	4	3	3	5
5	5	5	5	4	3	4	3	4
5	5	5	3	4	1	4	3	4
5	5	5	5	3	3	1	1	2
4	4	4	4	4	4	4	4	4
5	5	5	5	5	5	5	5	5
3	3	3	4	3	3	1	1	1
5	5	5	3	4	3	2	3	4
5	5	5	3	3	3	4	2	5
5	5	5	5	5	5	5	5	5
4	4	4	4	4	4	4	4	4
4	4	4	4	4	4	4	4	4
5	5	5	2	2	2	2	2	3
5	5	5	3	2	1	5	5	5
5	5	5	5	5	4	4	5	5
4	4	4	4	5	4	2	3	3
5	5	5	5	5	5	5	5	5
4	4	4	4	4	2	3	3	3
5	5	5	5	5	5	5	5	5
5	5	5	5	5	5	5	5	5
5	5	5	5	5	5	5	5	5
3	3	3	3	3	2	3	3	3
5	5	5	5	5	5	5	5	5
5	5	5	5	3	3	3	2	2
5	5	5	5	5	5	5	5	5
5	5	5	5	5	5	5	5	5
3	3	3	5	4	3	3	3	3
3	3	3	2	2	2	1	1	1
4	4	4	4	4	3	3	3	3

5	5	5	5	5	5	5	5	5
5	5	5	5	5	5	5	5	5
3	3	3	3	3	2	2	3	3
5	5	5	5	5	5	5	5	4
5	5	5	5	5	5	5	5	5
3	3	3	3	3	3	3	3	3
5	5	5	5	5	5	5	5	5
5	5	5	5	5	1	2	2	2
5	5	5	5	5	5	5	5	5
5	5	5	3	3	4	3	3	3
4	4	4	4	5	4	3	3	3
5	5	5	5	5	5	5	5	5
4	4	4	3	4	3	3	3	3
4	4	4	4	4	4	4	4	4
5	5	5	5	5	5	5	5	5
5	5	5	5	5	5	5	5	5
5	5	5	5	5	5	5	5	5
5	5	5	5	5	5	5	5	5
5	5	5	5	5	3	5	5	5
5	5	5	5	5	5	5	5	5
5	5	5	5	5	5	5	5	5
5	5	5	2	5	5	5	3	4
5	4	5	5	5	4	5	4	5
5	5	5	4	3	3	2	2	2
5	5	5	5	5	5	5	5	5
5	5	5	5	5	3	3	4	5
3	3	2	3	5	2	2	2	2
5	5	5	4	5	4	4	4	4
5	5	5	5	5	5	5	5	5
5	5	5	5	5	5	5	5	5
4	4	4	4	4	4	4	4	4
5	5	5	5	5	5	5	5	5
3	3	3	3	3	3	3	3	3
5	5	5	5	5	5	5	5	5
5	5	5	5	5	5	5	5	5
3	3	3	5	5	3	2	2	2
5	5	5	5	5	4	4	4	4
5	5	5	3	4	3	3	3	3
4	4	4	3	3	3	2	2	3
5	5	5	5	5	5	5	5	5
5	5	5	5	5	5	5	5	5
5	5	5	5	5	5	4	4	4
5	5	5	5	5	5	5	5	5
5	5	5	4	4	3	3	3	3
3	4	4	2	4	3	3	2	3
5	5	5	5	5	3	3	4	4
5	5	4	4	3	5	3	5	3
4	4	4	4	4	4	4	4	4
4	4	4	3	3	3	3	3	3
4	4	4	3	3	3	2	2	4
3	3	3	3	3	3	3	3	3
5	5	5	3	3	3	3	3	3
1	1	1	1	1	1	1	1	1
5	5	5	4	4	4	4	4	4

4	4	4	5	5	1	1	1	1
4	4	4	5	3	2	4	4	4
4	4	4	2	3	4	4	4	4
5	5	5	5	2	2	2	2	2
3	3	3	4	4	3	3	3	3
5	5	5	3	2	2	2	4	4
5	5	5	3	2	2	4	4	4
4	5	4	5	4	3	3	4	4
4	2	4	5	3	4	3	2	3
5	5	5	5	5	5	5	5	5
4	4	4	4	5	5	4	4	4
1	3	4	4	3	3	3	3	2
4	3	3	3	3	2	2	2	2
4	3	4	3	3	2	4	3	4
5	5	5	5	5	3	2	3	5
5	5	5	5	5	5	5	5	5
5	5	5	5	5	5	5	5	5
5	5	5	5	5	5	5	5	5
5	5	5	3	5	5	3	3	3
5	5	5	5	5	5	5	5	5
4	5	5	5	5	5	5	5	5
5	5	5	5	5	5	5	5	5
5	5	5	5	5	5	5	5	5
5	5	5	5	3	3	3	3	3
1	1	1	2	2	1	1	1	5
4	4	4	4	4	2	3	3	3
5	5	5	5	5	5	5	5	5
5	5	5	5	5	5	3	3	3
5	3	3	1	4	1	3	3	3
4	5	4	4	4	4	5	4	4
5	5	5	4	3	3	3	3	3
5	5	5	5	5	5	5	5	5
3	3	3	3	3	3	3	3	3
5	5	5	5	5	5	5	5	5
5	5	5	5	5	5	5	5	5
5	5	5	5	5	5	5	5	5
4	4	4	4	4	4	4	4	4
5	4	4	5	3	3	2	2	2
3	3	3	1	2	1	1	1	1
5	5	5	3	2	2	1	3	3
4	4	4	5	5	4	3	3	3
3	5	3	3	3	5	1	1	2
4	4	4	4	3	3	2	2	3
4	4	4	4	4	4	4	4	4
5	5	5	1	2	2	1	1	2
5	5	5	5	5	5	3	3	3
5	5	5	5	4	2	4	4	4
4	4	4	5	5	4	3	3	3
5	5	5	4	4	4	4	5	5
3	3	3	5	1	1	1	1	1
3	3	3	3	3	3	3	3	3
5	5	5	5	5	4	3	3	3
4	4	4	5	5	4	3	3	3
4	4	4	5	5	3	4	4	3

4	4	4	3	5	3	3	3	3
4	4	4	4	3	3	3	3	4
4	4	4	2	4	3	3	3	3
5	5	5	5	5	3	4	3	3
5	5	5	5	5	5	5	5	5
5	5	5	5	5	5	5	5	5
4	4	4	4	4	4	4	4	4
5	5	5	5	5	5	5	5	5
4	4	4	4	4	4	3	3	4
5	5	5	2	4	2	3	4	4
4	4	4	3	3	3	3	3	3
5	5	5	4	4	2	3	5	5
5	5	5	5	5	5	5	5	5
5	5	5	5	4	4	3	3	3
5	5	5	5	5	5	5	5	5
5	5	5	5	5	5	5	5	5
5	5	5	5	5	5	5	5	5
5	5	5	3	3	3	3	3	5
5	5	5	5	5	5	5	5	5
3	4	4	3	3	4	4	3	3
4	3	5	4	3	5	3	4	5
5	5	5	5	5	3	5	5	5
5	5	5	3	3	3	5	5	5
4	4	4	3	4	3	3	3	3
5	5	5	2	3	2	3	3	3
5	5	5	5	5	5	5	5	5
5	5	5	5	5	5	5	5	5
5	5	5	5	5	5	5	5	5
5	5	5	5	5	3	3	3	3
4	4	4	4	5	3	3	3	3
5	5	5	5	5	5	5	5	5
5	5	5	5	5	5	4	4	5
5	5	5	5	5	5	5	5	5
5	5	5	5	5	5	3	3	3
3	3	3	5	5	4	3	1	5
5	4	5	5	5	2	5	5	5
4	4	3	3	3	4	1	3	3
5	5	5	4	5	5	4	5	4
5	5	5	5	5	5	5	5	5
4	4	4	4	4	4	4	4	4
5	5	5	5	5	5	5	5	5
5	5	5	5	5	5	5	5	5
5	5	5	5	5	5	5	5	5
5	5	5	5	5	2	5	5	5
5	5	5	3	5	5	4	4	4
5	5	5	5	5	1	1	1	5
3	3	3	3	3	3	3	3	3
5	5	5	5	5	5	5	5	5
5	5	5	5	5	5	5	5	5
5	5	5	5	5	3	5	5	5
3	3	3	4	5	4	2	2	2
5	5	5	5	5	5	5	5	5
3	3	3	5	3	3	3	3	3
5	5	5	5	5	5	5	5	5

4	4	4	3	3	3	4	3	3
4	4	4	4	5	5	3	4	4
5	4	5	4	3	4	1	1	2
5	5	5	4	5	2	3	3	3
3	3	3	3	3	1	1	4	4
4	4	4	3	4	3	3	3	3
3	3	3	3	3	3	3	3	3
5	5	5	5	5	5	5	5	5
5	5	5	5	5	5	5	5	5
3	5	3	5	5	2	3	3	3
3	3	3	4	4	2	3	3	3
5	5	5	5	5	5	5	5	5
4	4	4	4	4	4	4	4	4
4	4	4	5	4	4	4	4	4
5	5	5	5	5	5	5	5	5
4	4	4	3	4	2	3	3	3
5	5	5	5	5	5	5	5	5
5	5	5	5	5	5	4	5	5
3	3	3	5	5	5	3	3	3
5	5	5	5	5	3	3	3	3
5	5	5	1	5	5	3	3	3
1	1	1	2	2	1	4	4	4
2	2	2	4	3	1	1	2	2
5	5	5	4	4	2	3	3	4
5	5	5	5	5	3	5	5	5
4	5	4	4	3	2	3	3	4
5	5	5	5	5	5	5	5	5
5	5	5	5	5	5	5	5	5
5	5	5	5	5	5	5	5	5
5	5	5	5	5	5	5	5	5
5	5	5	5	5	5	5	5	5
5	5	5	3	3	3	3	3	3
5	5	5	3	3	2	3	3	3
5	5	5	1	5	5	5	5	5
4	4	4	4	4	4	4	4	4
4	4	4	3	3	2	4	4	5
5	5	5	3	2	1	1	1	1
5	4	4	3	4	3	3	4	3
5	5	5	5	5	5	5	5	5
3	3	3	3	3	3	3	3	3
3	3	4	4	5	2	3	4	3
5	5	5	5	5	5	5	5	5
4	3	3	3	4	4	3	4	3
3	3	3	5	5	1	2	2	2
5	5	5	3	3	3	2	2	2
5	5	5	3	3	2	5	5	5
5	5	5	5	5	3	4	4	4
4	4	4	4	5	4	3	3	4
3	3	3	3	3	3	3	3	3
5	5	5	5	5	5	5	5	5
4	4	4	4	4	4	4	4	4
5	5	5	3	5	5	5	5	5
5	5	5	5	5	5	5	5	5
5	5	5	5	5	5	5	5	5

5	5	5	4	4	3	4	4	5
5	4	5	2	4	3	2	3	3
5	5	5	5	5	5	5	5	5
5	5	5	5	5	5	5	5	5
4	4	4	4	4	4	3	3	3
5	5	5	5	5	5	5	5	5
5	5	5	3	4	5	4	4	4
4	4	4	4	3	3	3	3	5
5	5	5	5	5	5	5	5	5
5	5	5	5	5	5	5	5	5
5	5	5	5	5	5	5	5	5
4	4	4	4	4	4	4	4	4
5	5	5	5	5	5	5	5	5
5	5	5	5	5	5	5	5	5
3	3	3	3	4	3	3	3	3
4	4	4	4	4	4	4	4	4
3	3	3	5	4	2	4	3	4
5	5	5	5	5	5	5	5	5
5	5	5	5	5	5	5	5	5
5	5	5	3	3	2	2	1	1
5	5	5	5	5	5	5	5	5
5	5	5	5	5	5	5	5	5
5	5	5	5	5	5	5	5	5
4	5	5	1	1	4	1	1	1
5	5	5	3	2	2	5	5	5
5	5	5	1	3	4	2	1	3
5	5	5	1	1	3	1	1	4
4	4	4	4	3	3	3	3	3
5	5	5	5	5	5	5	5	5
5	5	5	3	4	3	4	3	3
5	5	5	3	3	5	3	2	3
5	5	5	5	5	5	5	5	5
5	5	5	5	5	5	5	5	5
5	5	5	3	3	4	5	3	4
4	4	4	4	4	4	3	3	3
5	5	5	3	3	3	4	4	4
5	5	5	5	5	5	5	5	5
3	3	3	5	2	2	2	2	2
5	5	4	3	3	3	4	4	4
4	3	4	5	5	4	3	3	3
3	3	3	5	3	1	2	2	2
4	3	3	4	3	3	3	3	3
4	4	4	3	3	3	2	2	2
3	3	3	3	4	4	3	3	3
5	5	5	3	4	3	3	2	4
4	4	4	4	4	4	4	4	4
5	5	5	2	1	1	2	2	2
4	4	4	4	4	4	4	4	4
5	5	5	5	5	5	5	5	5
5	5	5	5	5	5	5	5	5
4	4	4	4	4	4	4	4	4
4	4	4	4	3	4	4	3	4
4	3	3	4	4	3	4	4	4
5	5	5	5	5	5	5	5	5

5	5	5	3	3	3	3	3	3
5	5	5	3	5	1	1	1	3
4	4	4	4	3	2	3	3	3
4	3	4	5	4	4	2	2	2
5	5	5	5	5	3	3	4	4
2	2	2	5	3	1	1	1	1
4	4	4	5	5	3	3	3	3
5	5	5	3	5	5	5	3	5
4	4	4	5	4	3	1	1	1
3	3	3	5	5	3	1	1	1
5	5	5	5	5	5	5	5	5
5	5	5	3	4	4	3	4	4
5	5	5	5	5	5	5	5	5
5	5	5	5	5	5	5	5	5
5	5	5	3	3	3	2	3	3
5	5	5	3	3	3	3	3	3
5	5	5	5	5	5	5	5	5
5	5	5	5	5	5	5	5	5
1	1	1	1	1	1	1	1	1
4	4	4	4	4	4	4	4	4
5	5	5	5	5	5	5	5	5
5	5	5	5	5	3	3	3	3
5	5	5	5	5	5	5	5	5
4	4	4	5	5	3	3	3	3
4	5	5	4	5	4	4	5	5
5	5	5	5	5	5	5	5	5
5	4	5	5	4	1	1	1	1
5	5	5	5	5	5	5	5	5
5	5	5	2	2	2	5	5	5

GPA1	GPA2	GPA3	GPA4	GPA5	Intentior	Intentior	Intentior	Intentior
5	5	5	5	5	5	5	5	5
4	4	4	4	4	4	4	4	4
4	4	4	4	4	3	3	3	4
5	5	5	5	5	5	5	5	5
4	4	4	4	4	4	4	4	3
5	5	5	5	5	3	3	5	4
5	5	5	5	5	5	5	5	5
3	3	3	3	3	3	3	3	3
5	4	3	3	5	3	2	2	2
5	5	5	5	5	5	5	5	5
3	3	3	3	3	3	3	3	3
4	4	4	4	4	4	4	4	4
5	5	5	5	5	5	5	5	5
5	5	5	5	5	5	5	4	5
5	5	5	5	5	5	5	5	5
4	3	4	3	3	4	4	3	4
5	5	5	5	5	5	5	5	5
5	5	5	5	5	5	5	5	5
4	4	4	4	4	4	4	4	4
5	5	5	5	5	5	5	5	5
5	5	5	5	5	5	4	5	4
5	5	5	5	5	5	5	5	5
5	5	5	5	5	5	5	5	5
5	5	5	5	5	5	5	5	5
5	5	2	3	5	5	5	5	5
3	3	3	3	3	3	3	3	3
5	5	5	5	5	5	3	4	4
3	3	3	3	3	3	3	3	3
5	5	5	5	5	5	5	5	5
4	4	4	4	4	4	4	4	4
5	5	3	4	4	4	4	5	5
5	5	5	5	5	3	2	3	3
5	5	5	5	5	5	5	5	5
5	5	5	5	5	5	5	5	5
5	5	4	5	5	5	4	4	4
4	4	4	5	5	4	4	4	4
4	4	3	3	4	3	3	3	3
3	4	4	4	4	4	4	4	4
5	5	5	5	5	5	5	5	5
5	5	5	5	5	5	5	5	5
5	5	5	5	5	5	5	5	5
2	3	3	3	2	3	3	3	2
4	4	4	5	5	4	5	4	5
5	5	5	5	5	5	5	5	5
5	5	5	5	5	5	5	5	5
5	5	5	5	5	5	5	5	5
5	5	5	5	5	5	3	1	3
5	5	5	5	5	3	3	5	5
5	5	5	5	5	3	3	4	4
5	5	5	5	5	5	5	5	5
5	5	5	5	5	5	5	5	5
5	5	5	5	5	5	5	5	5
5	5	5	5	5	5	5	5	5

4	5	4	4	3	4	4	3	3
5	5	5	5	5	5	4	5	5
4	4	4	4	4	4	4	4	4
4	4	4	4	4	4	5	3	4
5	5	5	5	5	5	5	5	5
5	5	3	4	4	4	3	4	4
5	5	5	5	5	5	4	5	5
5	5	5	5	5	5	5	5	5
5	5	5	5	5	5	5	5	5
5	5	4	5	5	5	3	3	5
4	4	3	3	3	3	3	3	3
4	4	4	4	4	4	4	4	4
5	5	5	5	5	5	5	5	5
5	5	5	5	5	5	5	5	5
5	5	5	5	5	5	5	4	5
5	5	5	5	5	5	5	3	4
5	5	5	5	5	5	5	5	5
5	5	5	5	5	5	5	5	5
4	5	5	5	5	4	4	5	3
5	5	5	5	5	5	5	5	5
5	5	5	3	5	5	5	5	5
5	5	5	5	5	5	5	5	5
5	5	5	5	5	5	5	5	5
5	5	5	5	5	5	5	5	5
4	4	4	4	4	5	3	3	3
4	5	5	5	5	5	5	4	5
5	5	5	4	5	5	5	5	5
5	5	3	2	5	3	3	4	5
5	5	5	5	5	5	5	5	5
4	4	4	4	4	4	4	4	3
5	5	5	5	5	5	5	5	5
2	4	2	3	3	3	3	3	2
5	5	5	5	5	5	5	5	5
5	5	4	3	3	5	3	5	4
5	5	5	5	5	5	5	5	5
4	4	4	4	5	4	4	4	4
4	4	4	4	4	4	4	4	4
4	4	4	4	4	5	5	5	5
5	5	5	5	5	5	5	5	5
5	5	5	5	5	5	5	5	4
3	4	4	4	4	4	3	4	4
5	5	5	5	5	5	5	5	5
4	4	4	4	4	4	4	4	4
5	5	5	5	5	5	5	5	5
5	5	5	5	5	5	5	5	5
5	5	5	5	5	5	5	5	5
3	3	3	3	3	3	3	3	3
5	5	5	5	5	5	5	5	5
4	4	4	4	4	4	3	3	3
5	5	5	5	5	5	5	5	5
5	5	5	5	5	5	5	5	5
5	5	5	4	5	3	3	5	5
4	4	3	3	3	3	2	2	2
5	5	4	5	5	4	5	5	4

5	5	5	5	5	5	5	5	5
5	5	5	5	5	5	5	5	5
3	3	3	3	3	3	3	3	3
5	5	5	5	5	5	5	5	5
5	5	5	5	5	5	5	5	5
3	3	3	3	3	3	3	3	3
5	5	5	5	5	5	5	5	5
3	3	4	4	4	3	3	3	3
5	5	5	5	5	5	5	5	5
5	5	5	5	5	4	4	4	3
4	4	4	4	4	4	3	5	5
5	5	5	5	5	5	5	5	5
3	3	3	3	3	4	4	4	4
4	4	4	4	4	4	4	4	4
5	5	5	5	5	5	5	5	5
5	5	5	5	5	5	5	5	5
5	5	5	5	5	5	5	5	5
5	5	5	5	5	5	5	5	5
5	5	5	5	5	5	5	5	5
5	5	5	5	5	5	5	5	5
5	5	5	5	5	5	5	5	5
5	5	5	5	5	5	5	5	5
5	5	5	5	5	5	5	5	5
4	5	4	4	5	4	4	4	4
5	5	5	5	5	5	5	5	5
5	5	5	5	5	5	5	5	5
4	4	3	4	4	3	4	4	5
5	5	5	5	5	4	4	4	4
5	5	5	5	5	5	5	5	5
5	5	5	5	5	5	5	5	5
4	4	4	4	4	4	4	4	4
5	5	5	5	5	5	5	5	5
3	3	3	3	3	3	3	3	3
5	5	5	5	5	5	5	5	5
5	5	5	5	5	5	5	5	5
3	3	3	3	5	3	3	4	3
5	5	5	5	5	5	5	5	5
5	5	5	4	5	4	3	3	3
5	5	5	5	5	5	5	5	5
5	5	5	5	5	5	5	5	5
5	5	5	5	5	5	5	5	5
4	4	4	4	4	4	4	4	4
5	5	5	5	5	5	5	5	5
3	3	5	5	5	5	5	5	5
3	4	4	4	4	5	5	3	4
4	4	4	4	4	4	3	4	3
5	5	4	3	4	4	4	2	5
4	4	4	4	4	4	4	4	4
5	5	5	5	5	4	4	4	4
4	4	3	4	4	4	4	3	4
3	3	3	3	3	3	3	3	3
3	3	4	4	5	4	4	4	4
1	1	1	1	1	1	1	1	1
4	4	4	4	4	4	4	4	4

4	4	4	4	4	4	4	4	4
5	5	5	5	5	5	5	5	5
5	3	5	5	3	3	3	3	3
4	4	4	4	4	3	3	3	3
3	4	3	3	3	3	4	3	2
5	5	5	3	3	3	2	2	2
5	5	5	5	5	5	4	5	4
4	5	5	4	5	5	3	4	4
3	3	1	3	3	3	3	3	3
5	5	5	5	5	5	5	5	5
5	5	4	3	3	4	4	4	3
3	3	4	3	3	3	3	3	3
4	4	3	3	2	3	2	3	2
5	5	4	4	5	4	4	5	5
5	5	5	5	5	5	5	5	5
5	5	5	5	5	5	5	5	5
5	5	5	5	5	5	5	5	5
5	5	5	5	5	5	5	5	5
3	3	3	3	3	5	4	5	5
5	5	5	5	5	5	5	5	5
5	5	5	5	5	5	5	5	5
5	5	5	5	5	5	5	5	5
5	5	5	5	5	5	5	5	5
4	4	3	3	3	3	3	3	3
1	1	1	1	1	1	1	1	1
3	3	3	3	3	3	3	3	3
5	5	5	5	5	5	5	5	5
5	5	5	5	5	5	5	5	5
3	3	5	5	3	3	3	3	3
5	5	4	5	4	5	5	5	5
5	5	5	5	5	5	3	3	3
5	5	5	5	5	5	5	5	5
3	3	3	3	3	3	3	3	3
5	5	5	5	5	5	5	5	5
5	5	5	5	5	5	5	5	5
5	5	5	5	5	5	5	5	5
4	4	4	4	4	4	4	4	4
3	5	5	4	5	5	4	3	3
4	4	3	3	4	3	1	3	1
4	4	5	3	5	5	5	5	5
4	4	4	3	3	3	3	3	3
5	5	3	4	5	3	4	4	4
4	4	4	3	4	4	3	4	4
4	4	4	4	4	4	4	4	4
5	5	5	3	3	4	4	4	4
5	5	5	5	5	5	5	5	5
3	3	4	4	3	4	3	4	4
5	5	5	5	5	5	4	4	5
5	5	5	5	5	5	5	5	5
5	5	1	3	5	1	1	1	1
3	3	3	3	3	3	3	3	3
5	5	5	5	5	5	5	5	5
3	4	3	4	4	3	3	3	3
5	5	4	4	5	4	3	4	4

4	3	4	3	4	4	4	4	4
5	5	4	4	5	4	4	4	4
5	4	4	4	5	4	4	3	4
5	5	5	5	5	5	4	4	5
5	5	5	5	5	5	5	5	5
5	5	5	5	5	5	5	5	5
4	4	4	4	4	4	4	4	4
5	5	5	5	5	5	5	5	5
4	4	3	4	4	4	4	3	3
5	5	5	5	5	5	5	5	5
3	3	3	3	3	3	3	3	3
5	5	5	5	5	5	4	5	5
5	5	5	5	5	5	5	5	5
4	4	4	4	4	5	4	4	4
5	5	5	5	5	5	5	5	5
5	5	5	5	5	5	5	5	5
5	5	5	5	5	5	5	5	5
5	5	5	5	5	5	5	5	5
5	5	5	5	5	5	5	5	5
4	3	3	4	3	4	3	4	3
5	5	4	4	4	5	4	5	4
5	5	5	5	5	5	5	5	5
4	4	4	4	5	4	5	4	5
5	5	5	5	5	4	4	4	4
5	5	5	5	5	5	5	5	5
5	5	5	5	5	5	5	5	5
5	5	5	5	5	5	5	5	5
5	5	5	5	5	5	5	5	5
5	5	5	5	5	5	4	4	5
4	5	5	5	4	4	3	4	4
5	5	5	5	5	5	5	5	5
5	5	5	5	5	5	5	5	5
5	5	5	5	5	5	5	5	5
5	5	5	5	5	5	5	5	5
5	5	5	5	5	5	5	3	2
5	5	5	5	5	5	5	5	5
2	1	2	3	2	2	3	2	1
5	5	4	4	4	4	4	5	5
5	5	5	5	5	5	5	5	5
4	4	4	4	4	4	4	4	4
5	5	5	5	4	5	5	5	5
5	5	5	5	5	5	5	5	5
5	5	5	5	5	5	5	5	5
5	5	5	5	5	5	5	5	5
4	4	4	4	4	4	3	3	5
3	3	5	4	5	5	4	5	5
3	3	3	3	3	3	3	3	3
5	5	5	5	5	5	5	5	5
5	5	5	5	5	5	5	5	5
5	5	5	5	1	5	5	5	5
5	5	4	5	5	4	4	4	3
5	5	5	5	5	5	5	5	5
3	3	3	3	3	3	3	3	3
5	5	5	5	5	5	5	5	5

4	4	4	4	4	5	5	5	5
5	5	5	4	5	4	5	4	4
3	4	4	4	3	3	4	4	4
4	4	4	4	4	4	4	4	4
4	4	4	3	3	4	3	3	2
4	4	4	4	4	4	4	4	4
3	3	3	3	3	3	3	3	3
5	5	5	5	5	5	5	5	5
5	5	5	5	5	5	5	5	5
5	5	5	3	5	5	3	5	2
5	5	5	4	4	4	3	5	3
4	4	4	4	4	5	5	5	4
4	4	4	4	4	4	4	4	4
5	5	5	5	5	5	5	5	5
5	5	5	5	5	5	5	5	5
3	3	3	3	3	3	3	3	3
5	5	5	5	5	5	5	5	5
5	5	5	5	5	5	5	5	5
4	4	3	3	5	3	3	3	4
5	5	5	5	5	5	5	5	5
5	5	5	5	5	5	5	5	5
3	3	2	2	2	2	2	2	2
5	5	3	3	5	3	2	2	3
4	4	4	4	4	4	4	4	4
5	5	5	5	5	5	5	5	5
5	5	5	5	5	4	4	4	4
5	5	5	5	5	5	5	5	5
5	5	5	5	5	5	5	5	5
5	5	5	5	5	5	5	5	5
5	5	5	5	5	5	5	5	5
5	5	5	5	5	5	5	5	5
5	5	5	5	5	5	3	5	5
5	5	5	5	5	5	4	4	4
5	5	5	5	5	5	5	5	5
4	4	4	4	4	4	4	4	4
4	4	4	4	4	4	4	5	4
4	5	5	5	5	3	2	4	5
4	5	4	4	4	4	4	4	4
5	5	5	5	5	5	5	5	5
3	3	3	3	3	3	3	3	3
5	5	4	4	5	5	5	4	4
5	5	5	5	5	5	5	5	5
4	4	4	4	4	4	4	4	4
3	3	3	3	2	3	3	3	2
5	5	5	5	5	5	5	5	5
5	5	5	5	5	5	5	5	5
5	5	5	5	5	5	5	5	5
5	5	5	5	5	5	4	4	4
3	3	3	3	3	3	3	3	3
5	5	5	5	5	5	5	5	5
4	4	4	4	5	4	4	4	4
5	5	5	5	5	5	5	5	5
5	5	5	5	5	5	5	5	5
5	5	5	5	5	5	5	5	5

4	4	5	5	5	5	5	5	5
4	3	4	4	3	4	5	3	3
5	5	5	5	5	5	5	5	5
5	5	5	5	5	5	5	5	5
5	5	5	5	5	4	4	4	4
5	5	5	5	5	5	5	5	5
5	5	5	5	5	5	5	5	5
5	5	5	5	5	5	5	5	4
5	5	5	5	5	5	5	5	5
5	5	5	5	5	5	5	5	5
5	5	5	5	5	5	5	5	5
4	4	4	4	4	4	4	4	4
5	5	5	5	5	5	5	5	5
5	5	5	5	5	5	5	5	5
4	4	4	4	4	3	3	3	3
5	5	5	4	4	4	4	4	4
3	3	4	4	3	5	5	4	4
5	5	5	5	5	5	5	5	5
5	5	5	5	5	5	5	5	5
5	5	5	5	5	5	5	5	5
5	5	5	5	5	5	5	5	5
5	5	5	5	5	5	5	5	5
5	5	5	5	5	5	5	5	5
5	5	3	3	5	5	4	1	1
5	5	5	5	5	5	5	3	3
5	5	5	5	5	5	3	3	5
5	5	5	5	5	4	4	3	4
4	4	2	3	3	3	3	3	3
5	5	5	5	5	5	5	5	5
5	5	5	5	5	5	5	5	5
5	5	5	5	5	5	5	4	4
5	5	5	5	5	5	5	5	5
5	5	5	5	5	5	5	5	5
5	5	5	5	5	5	4	5	5
3	3	3	3	3	3	3	3	3
5	5	5	5	5	5	5	5	5
5	5	5	5	5	5	5	5	5
3	3	3	3	3	3	3	3	3
5	5	5	5	5	5	5	5	5
3	4	2	4	5	3	2	4	2
4	4	4	3	4	3	3	3	3
4	4	4	4	4	3	3	4	3
5	5	5	5	5	4	4	4	4
3	4	3	3	3	3	3	3	4
5	5	5	5	5	5	4	4	4
4	4	4	4	4	4	4	4	4
4	3	5	5	5	3	3	3	4
4	4	4	4	4	4	4	4	4
5	5	5	5	5	5	5	5	5
5	5	5	5	5	5	5	5	5
4	5	5	5	5	4	5	5	5
3	4	3	4	4	4	3	3	4
3	3	3	3	4	4	4	3	2
5	5	5	5	5	5	5	5	5

5	5	5	5	5	5	4	5	5
3	5	5	3	3	5	1	3	1
4	4	4	4	4	4	4	5	4
3	3	3	4	4	3	2	2	2
5	5	2	4	3	4	4	4	2
3	2	2	2	2	1	1	4	1
5	5	5	5	5	3	3	3	3
5	5	5	5	5	5	4	5	3
4	4	3	3	3	2	3	3	2
3	3	3	3	3	3	3	3	3
5	5	5	5	5	5	5	5	5
4	4	4	4	4	4	3	4	4
5	5	5	5	5	5	5	5	5
5	5	5	5	5	5	5	5	5
3	3	3	3	3	3	3	3	3
5	5	5	5	5	4	3	3	3
5	5	5	5	5	5	5	5	5
5	5	5	5	5	5	5	5	5
1	1	1	1	1	1	1	1	1
4	4	5	3	3	4	3	3	3
5	5	5	5	5	5	5	5	5
5	5	5	5	5	5	5	5	5
5	5	5	5	5	5	5	5	5
5	5	4	4	4	4	4	4	4
4	5	5	5	5	5	5	5	5
5	5	5	5	5	5	5	5	5
2	2	3	2	2	2	2	2	2
5	5	5	5	5	5	5	5	5
5	5	5	5	5	5	5	5	5

BV1	BV2	BV3	BV4	AV1	AV2	AV3	AV4	AV5
5	5	5	5	5	5	5	5	5
5	4	5	5	4	4	3	2	4
5	5	5	5	5	5	5	1	4
5	5	5	5	5	5	5	2	2
5	5	5	5	4	4	4	4	4
5	5	5	5	5	5	5	5	5
5	5	5	5	5	5	4	5	1
5	5	5	5	3	3	3	3	3
5	4	5	5	5	5	5	5	4
5	5	5	5	5	5	5	1	5
3	3	3	3	3	3	3	3	3
4	4	4	4	4	4	4	4	4
5	5	5	5	5	5	3	3	5
5	5	5	5	5	5	5	5	2
5	5	5	5	5	5	5	5	5
5	5	5	5	5	4	3	3	5
5	5	5	5	5	5	5	5	5
5	5	5	5	5	3	4	5	1
4	4	4	4	4	4	4	4	4
5	5	5	5	1	1	1	1	1
5	5	5	5	5	5	1	3	3
5	5	5	5	5	5	5	5	5
5	5	5	5	5	1	5	5	1
5	5	5	5	5	5	5	1	1
5	5	5	5	5	3	3	4	2
3	3	3	3	3	3	3	2	2
5	5	5	5	5	2	2	1	1
5	5	5	5	5	5	4	3	5
5	5	5	5	1	1	1	1	1
4	4	4	4	4	4	4	4	4
5	5	5	5	5	5	4	4	5
5	4	5	5	5	3	3	4	4
5	5	5	5	4	4	4	4	4
5	5	5	5	5	5	5	5	5
5	5	5	5	5	3	5	5	5
5	5	5	5	5	5	5	5	5
5	5	5	5	5	3	5	1	4
5	5	5	5	5	5	5	3	5
5	5	5	5	5	5	5	5	5
5	5	5	5	5	5	5	5	5
5	5	5	5	5	5	5	5	5
4	4	5	4	5	4	4	3	3
4	4	4	4	5	2	5	5	5
5	5	5	5	5	5	5	5	5
5	5	5	5	5	5	5	2	4
5	5	5	5	5	5	5	5	5
5	5	5	5	5	3	3	1	2
5	5	5	5	3	3	5	5	5
5	5	5	5	5	1	2	1	3
5	5	5	5	5	5	5	5	3
5	5	5	5	5	5	3	5	1
5	5	5	5	5	5	5	5	5
5	5	5	5	5	5	5	5	5

5	5	5	5	5	5	5	3	5
5	5	5	5	3	5	5	1	4
4	4	4	4	4	4	4	4	4
5	4	5	5	3	4	4	3	4
5	5	5	5	5	5	5	5	5
5	4	5	5	4	4	4	1	4
5	5	5	5	5	5	5	1	5
5	5	5	5	5	5	5	1	5
5	5	5	5	5	5	5	5	5
5	5	5	5	5	5	5	3	4
3	3	3	3	4	4	4	1	4
5	5	5	5	4	3	2	2	3
5	5	5	5	5	5	5	5	5
5	5	5	5	5	5	5	5	5
5	5	5	5	3	3	2	1	3
5	5	5	5	4	2	2	2	5
5	5	5	5	5	5	5	5	5
5	5	5	5	5	5	5	5	5
5	5	5	5	5	4	5	5	5
5	5	5	5	5	5	5	5	5
5	5	5	5	5	2	1	3	4
5	5	5	5	5	5	5	5	3
5	5	5	5	5	5	4	5	3
5	5	5	5	4	3	3	1	2
5	2	5	5	5	5	4	5	5
5	4	5	5	5	5	5	5	5
5	5	5	5	5	3	4	5	5
5	4	5	5	5	5	5	5	3
5	5	5	5	2	4	3	1	1
4	4	4	4	4	4	4	3	3
5	5	5	5	5	5	5	2	5
5	5	5	5	4	4	4	1	4
5	3	5	5	5	5	5	5	5
5	5	5	5	5	5	3	1	1
5	5	5	5	3	2	1	3	4
4	4	4	4	4	4	5	3	3
4	4	4	4	5	1	1	1	1
5	5	5	5	1	1	1	1	1
5	5	5	5	5	5	4	2	5
5	5	5	5	5	3	5	1	5
4	4	5	5	5	5	5	1	5
5	5	5	5	5	5	5	5	5
4	4	4	4	4	3	4	1	3
5	5	5	5	5	5	5	5	5
5	5	5	5	5	5	5	5	5
5	5	5	5	5	5	5	5	5
5	5	5	5	3	2	3	3	2
5	5	5	5	4	1	1	1	1
5	4	5	5	5	3	3	1	3
5	5	5	5	5	5	5	5	5
5	5	5	5	5	5	5	5	5
5	5	5	5	5	5	5	5	5
5	5	5	5	3	2	3	1	1
5	5	5	5	5	4	4	1	5

5	5	5	5	5	5	5	5	5
5	5	5	5	5	5	5	5	5
3	3	3	3	3	3	3	3	3
5	5	5	5	2	2	4	1	4
5	5	5	5	5	5	5	3	5
5	5	5	5	5	5	5	5	5
5	5	5	5	5	3	3	2	3
4	4	4	4	5	5	5	1	5
5	5	5	5	4	4	4	3	5
5	5	5	5	5	5	5	3	4
5	5	5	5	4	4	4	4	4
5	5	5	5	5	1	2	2	5
4	4	4	4	4	4	4	4	4
4	4	4	4	4	4	4	4	4
5	5	5	5	5	5	5	5	5
5	5	5	5	5	5	5	5	5
5	5	5	5	5	5	5	5	5
5	5	5	5	5	5	5	5	5
5	5	5	5	5	3	2	2	5
5	5	5	5	5	4	5	4	5
5	5	5	5	5	4	2	1	4
5	5	5	5	5	5	5	2	5
5	5	5	5	3	5	5	3	4
5	5	5	5	5	3	5	3	5
5	5	5	5	5	2	5	2	5
5	5	5	5	3	2	2	5	5
5	5	5	5	4	2	3	5	4
5	5	5	5	3	4	4	2	4
5	5	5	5	5	5	5	5	5
5	5	5	5	5	5	5	5	5
4	4	4	4	4	4	4	4	4
5	5	5	5	5	5	5	5	5
3	3	3	3	3	3	3	3	3
5	5	5	5	5	5	5	5	5
5	5	5	5	5	2	3	3	1
5	5	5	5	5	5	5	5	5
5	5	5	5	5	5	5	5	5
5	5	5	5	5	4	3	5	3
5	5	5	5	2	1	1	1	1
5	5	5	5	5	5	5	5	5
5	5	5	5	5	5	5	5	5
4	4	4	4	4	4	4	4	4
5	5	4	5	5	5	5	5	5
5	5	5	5	5	5	5	5	5
3	4	4	3	5	4	1	1	5
5	4	5	5	5	5	4	3	3
5	3	5	3	5	3	3	3	3
4	4	4	4	4	4	4	4	4
5	5	5	5	4	3	2	2	3
5	5	5	5	5	5	4	5	5
3	3	3	3	3	3	4	4	4
5	5	5	5	5	5	5	5	5
1	1	1	1	1	1	1	1	1
5	5	5	5	3	3	4	4	5

5	5	5	5	5	5	5	5	5
5	5	5	5	5	3	5	1	5
5	3	5	5	5	1	1	2	5
5	5	5	5	4	3	1	4	1
4	3	5	4	5	3	5	1	2
5	5	5	5	3	3	2	2	5
5	5	5	5	5	4	1	2	1
4	5	5	5	5	5	1	1	5
5	5	5	5	4	5	4	3	4
5	5	5	5	5	5	5	5	5
5	5	5	5	2	3	2	3	4
5	5	5	5	5	4	4	4	4
5	5	5	5	4	4	4	2	4
4	4	3	3	4	3	4	3	3
5	5	5	5	5	5	5	5	5
5	5	5	5	5	5	5	5	5
5	5	5	5	5	5	5	5	5
5	5	5	5	5	5	5	5	5
5	5	5	5	5	5	5	5	5
5	5	5	5	5	5	5	5	5
5	5	5	5	5	5	5	5	5
5	5	5	5	5	5	5	5	5
5	5	5	5	5	5	5	5	5
5	4	5	5	4	3	4	4	5
1	1	1	1	1	1	1	1	1
5	5	5	5	3	2	2	2	2
5	5	5	5	5	5	5	5	5
5	5	5	5	5	5	5	5	5
3	4	5	5	4	3	1	1	2
5	4	5	5	3	4	2	2	5
5	4	5	5	5	3	1	5	5
5	5	5	5	3	4	4	1	4
5	5	5	5	3	3	3	3	3
5	5	5	5	5	5	5	5	5
5	5	5	5	3	3	3	3	5
5	5	5	5	5	5	5	5	5
5	5	5	5	5	3	1	1	4
5	5	5	5	5	5	5	1	5
5	5	5	5	5	5	5	5	5
5	5	5	1	5	5	3	5	5
5	5	5	3	5	5	5	1	1
5	5	5	5	5	1	5	1	5
5	4	5	5	3	3	3	3	4
4	4	4	4	4	4	4	4	4
5	4	5	4	5	3	2	1	5
5	5	5	5	1	4	1	3	5
4	3	5	3	4	4	4	4	4
5	5	5	5	5	5	5	5	5
5	5	5	5	5	3	5	1	1
5	5	5	5	5	5	5	1	5
3	3	3	3	3	3	3	3	3
5	5	5	5	5	5	5	5	5
3	3	3	4	3	3	3	3	3
5	5	5	5	4	5	2	3	5

5	5	5	5	4	4	4	4	5
5	5	5	5	5	5	4	4	3
5	5	5	5	5	5	5	5	5
5	5	5	5	5	4	2	3	4
5	5	5	5	5	5	5	5	5
5	5	5	5	5	5	5	5	5
4	4	4	4	4	4	4	4	4
5	5	5	5	5	5	5	5	5
4	4	4	4	5	5	5	3	5
5	5	5	5	5	3	4	3	5
5	4	5	4	5	3	3	4	4
5	5	5	5	5	5	5	5	5
5	5	5	5	5	5	5	3	3
5	4	5	5	5	5	5	5	5
5	5	5	5	5	5	5	5	5
5	5	5	5	5	5	5	5	5
5	5	5	5	5	5	5	5	5
5	5	5	4	5	5	5	1	5
5	5	5	5	5	5	5	5	5
4	4	3	4	3	4	4	3	4
5	5	4	5	4	3	4	3	5
5	5	5	5	5	3	5	1	5
4	4	5	5	3	3	3	3	4
5	4	5	4	3	3	4	1	5
5	5	5	5	5	3	2	2	5
5	5	5	5	5	5	5	5	5
5	5	5	5	5	5	5	1	5
5	5	5	5	5	5	5	5	5
5	5	5	5	3	2	2	3	3
5	4	5	5	4	5	5	4	3
5	5	5	5	1	1	2	1	5
5	5	5	5	5	5	5	5	5
5	5	5	5	5	3	5	5	5
5	5	5	5	5	1	1	5	1
5	5	5	5	4	4	1	2	1
5	5	5	5	5	4	4	5	5
5	5	5	5	4	5	5	1	3
5	4	4	4	5	5	5	5	5
5	5	5	5	5	5	5	5	5
4	4	4	4	4	4	4	4	4
5	5	5	4	4	4	2	4	5
5	5	5	5	5	5	5	5	5
5	5	5	5	5	5	5	5	5
5	5	5	5	5	5	4	5	5
5	5	5	5	4	5	5	5	5
5	5	5	5	5	1	1	1	3
5	5	5	5	3	3	3	3	3
5	5	5	5	5	5	5	5	5
5	5	5	5	5	5	5	5	5
5	5	5	5	5	1	1	1	3
5	5	5	5	5	3	5	1	1
5	5	5	5	5	2	4	1	5
3	3	3	3	3	3	3	3	3
5	5	5	5	5	5	5	5	5

5	5	5	5	5	5	5	5	5
5	5	5	5	4	4	4	4	4
5	5	5	5	4	4	4	2	4
5	5	5	5	5	5	5	1	3
5	3	5	3	4	1	2	1	3
5	4	5	5	5	5	5	1	5
3	3	3	3	3	3	3	3	3
5	5	5	5	3	3	3	1	1
5	5	5	5	5	5	3	1	5
5	5	5	5	3	1	2	1	5
5	4	5	5	5	5	5	5	5
4	4	4	4	4	4	5	5	5
4	4	5	5	5	5	5	5	5
5	5	5	5	5	5	5	5	5
5	5	5	5	5	5	5	5	5
4	4	4	4	4	1	3	3	3
5	5	5	5	5	5	5	5	5
5	5	5	5	5	5	5	3	5
5	5	5	5	5	5	3	1	4
5	5	5	5	3	1	1	1	3
5	5	5	5	5	5	5	5	5
5	5	5	5	3	3	3	1	5
5	5	5	5	5	3	3	2	3
5	5	5	5	5	5	5	5	5
5	5	5	5	5	5	4	2	5
5	5	5	5	4	2	3	2	3
5	5	5	5	5	5	5	5	5
5	5	5	5	5	5	5	5	5
5	5	5	5	5	5	5	5	5
5	5	5	5	4	4	4	4	4
5	5	5	5	5	5	5	5	5
5	5	5	5	5	5	5	3	3
5	5	5	5	5	3	2	2	3
5	5	5	5	5	5	5	5	5
4	4	4	4	3	2	3	2	2
5	5	5	5	4	3	4	5	3
5	5	5	5	5	5	5	1	4
5	4	5	3	4	3	2	1	1
5	5	5	5	5	5	5	5	5
5	5	5	5	5	3	5	1	5
5	5	5	5	5	4	5	3	5
5	5	5	5	5	5	5	5	5
5	5	5	5	3	3	2	1	2
5	4	5	5	5	1	4	3	3
5	5	5	5	5	5	5	3	5
5	5	5	5	3	4	3	3	5
5	5	5	5	5	3	3	3	3
5	5	5	5	5	1	5	3	4
3	3	3	3	3	3	3	3	3
5	5	5	5	5	3	3	3	3
4	4	5	5	4	4	4	4	4
5	5	5	5	5	5	5	5	5
5	5	5	5	5	5	5	5	5
5	5	5	5	5	5	5	5	5

5	4	5	5	4	4	5	3	5
4	5	5	5	5	1	4	1	4
5	5	5	5	5	5	3	1	3
5	5	5	5	5	5	5	5	5
5	4	5	5	3	4	3	1	4
5	5	5	5	5	5	5	5	5
5	5	5	5	5	4	3	1	3
5	5	5	5	4	4	4	3	4
5	5	5	5	5	5	5	5	5
5	5	5	5	5	5	5	5	5
5	5	5	5	5	5	5	5	5
4	4	4	4	4	4	4	4	4
5	5	5	5	5	5	5	5	5
5	5	5	5	5	5	5	5	5
4	3	4	4	4	4	5	2	3
5	5	5	5	4	4	3	2	4
2	3	4	4	5	4	5	3	3
5	5	5	5	5	5	5	5	5
5	5	5	5	5	5	5	5	5
5	5	5	5	5	5	5	5	5
5	5	5	5	5	5	5	5	5
5	5	5	5	5	5	5	5	5
5	5	5	5	5	5	5	1	5
5	5	5	5	5	5	5	1	5
5	2	5	2	5	5	5	1	1
5	5	5	5	3	3	3	1	4
5	5	5	5	4	3	5	1	1
5	3	5	5	3	1	2	5	5
5	5	5	5	5	5	5	5	5
5	5	5	5	5	5	5	3	3
5	5	5	5	5	2	5	5	5
5	5	5	5	5	5	5	5	5
5	5	5	5	5	5	5	5	5
5	5	5	5	5	5	5	4	5
5	4	5	4	5	3	3	4	4
5	5	5	5	5	5	3	3	3
5	5	5	5	5	5	5	5	5
5	5	5	5	4	4	4	3	4
5	5	5	5	5	5	5	2	2
5	5	5	5	5	5	1	1	5
5	5	5	5	4	5	5	3	4
4	4	4	4	4	2	2	2	4
5	5	5	5	4	4	4	1	5
4	4	5	5	3	3	1	1	3
5	5	5	5	5	5	5	5	5
4	4	4	4	3	3	1	1	1
5	5	5	5	2	3	2	1	1
4	4	4	4	4	4	4	4	4
5	5	5	5	5	5	5	5	5
5	5	5	5	5	5	5	5	5
4	5	5	5	5	5	4	4	5
4	3	3	4	4	4	3	4	3
4	3	5	4	3	4	2	1	2
5	5	5	5	5	5	5	5	5

5	5	5	5	4	4	4	4	4
5	5	5	5	5	1	3	1	5
5	5	5	5	4	3	3	2	5
5	3	5	4	4	3	4	2	3
5	4	5	4	5	5	5	5	5
5	2	5	5	4	1	1	1	3
5	5	5	5	4	3	3	3	4
5	5	5	5	2	2	3	2	2
5	4	5	4	5	3	5	1	1
5	5	5	5	4	4	2	1	3
5	5	5	5	5	5	5	5	5
5	5	5	5	5	4	3	3	3
5	5	5	5	5	5	5	5	5
5	5	5	5	5	5	5	5	5
5	3	3	5	5	5	3	3	3
5	5	5	5	3	5	5	4	1
5	5	5	5	5	5	5	5	5
5	5	5	5	5	5	5	5	5
5	5	5	5	5	5	5	5	5
5	5	5	5	3	3	4	2	3
5	5	5	5	5	5	5	5	5
5	5	5	5	5	5	5	5	5
5	5	5	5	5	3	5	1	5
5	5	5	5	5	4	4	4	4
5	5	5	5	4	4	5	5	5
5	5	5	5	5	5	5	5	5
5	3	5	5	2	2	2	2	2
5	5	5	5	5	5	5	3	3
5	5	5	5	5	5	5	5	5

AV6	CV1	CV2	CV3	CV4	CV5	AC1	AC2	AC3
5	5	5	5	5	5	2	2	3
4	4	4	4	4	4	3	4	4
4	4	5	5	5	5	5	5	5
3	3	4	3	2	3	4	4	3
4	5	5	5	5	5	5	5	5
5	5	5	5	5	5	5	5	5
5	3	3	3	3	3	2	2	2
3	3	3	3	3	3	3	3	3
5	5	5	5	5	5	3	2	1
5	5	5	5	5	5	5	5	5
3	3	3	5	3	5	3	3	3
4	4	4	4	4	4	4	4	4
5	5	5	5	5	5	5	5	5
5	5	5	5	5	5	3	5	2
5	5	5	5	5	5	5	5	5
5	5	5	5	5	5	5	5	5
5	5	5	5	5	5	5	5	5
5	5	3	4	4	4	5	5	5
4	4	4	4	4	4	4	4	4
1	5	5	5	5	5	5	5	5
5	5	5	5	5	5	1	3	3
5	5	5	5	5	5	5	5	5
5	5	5	5	5	5	2	2	1
5	5	5	5	5	5	2	2	2
5	5	5	5	4	5	2	3	3
2	5	5	5	3	5	3	3	3
4	3	3	3	3	3	3	3	3
5	4	3	3	3	3	4	5	4
1	5	5	5	5	5	5	5	4
4	4	4	4	4	4	4	4	4
4	5	5	5	3	5	4	4	4
4	4	4	5	5	3	3	3	2
4	4	4	4	4	4	3	4	4
5	5	5	5	5	5	5	5	5
5	5	5	5	5	5	3	3	4
5	5	5	5	5	5	5	5	5
4	5	5	5	5	5	3	4	3
5	5	5	5	5	5	5	5	5
5	5	5	5	5	5	5	5	5
5	5	5	5	5	5	5	5	5
5	5	5	5	5	5	5	5	5
4	4	4	4	4	5	3	3	3
5	5	5	4	5	4	5	5	5
5	5	5	5	5	5	5	5	5
5	5	5	5	5	5	5	5	5
5	5	5	5	5	5	3	2	3
5	5	5	5	5	3	4	4	4
5	5	5	5	5	5	5	5	5
4	5	5	5	5	5	5	5	5
5	5	5	5	5	5	5	5	5
5	5	5	5	5	5	5	5	5
5	5	5	5	5	5	5	5	5
5	5	5	5	5	5	4	5	4

5	5	5	5	5	5	4	4	3
3	5	5	4	3	4	2	3	4
4	4	4	4	4	4	5	5	5
4	4	4	4	5	4	3	4	4
5	5	5	5	5	5	5	5	5
4	4	4	4	4	5	5	4	4
5	5	5	5	5	5	4	4	5
5	5	5	5	5	5	1	3	5
5	5	5	5	5	5	5	5	5
5	5	5	5	5	5	5	5	5
5	4	4	4	4	4	4	4	4
4	3	4	4	4	4	4	4	4
5	5	5	5	5	5	5	5	5
5	5	5	5	5	5	5	5	5
5	3	5	5	5	5	3	4	4
4	4	3	4	5	4	2	2	2
5	5	5	5	5	5	5	5	5
5	5	5	5	5	5	5	5	5
5	5	5	5	5	5	5	5	5
5	5	5	5	5	5	2	4	3
5	5	5	5	3	5	3	3	5
5	5	3	5	4	4	5	5	5
5	5	4	5	5	3	4	4	4
2	5	5	5	5	5	5	5	5
5	5	5	4	3	1	3	4	3
5	5	5	5	5	5	4	4	4
5	5	5	5	5	5	4	3	5
5	5	5	5	5	5	3	3	2
4	4	4	5	5	3	2	3	3
4	3	4	4	4	4	3	3	3
5	5	5	5	5	5	5	5	5
5	4	4	4	4	5	3	3	3
5	5	5	5	5	5	3	3	3
5	5	3	5	4	3	3	5	2
3	3	3	3	3	3	3	3	3
4	3	4	4	4	4	4	4	3
4	4	4	4	4	4	4	4	3
1	4	4	4	3	3	2	2	2
5	5	5	5	5	5	3	5	4
4	4	5	5	5	4	3	5	3
5	5	5	5	5	5	3	3	3
5	5	5	5	5	5	5	5	5
4	4	4	4	4	4	3	3	4
5	5	5	5	5	5	5	5	5
5	5	5	5	5	5	5	5	5
5	5	5	5	5	5	5	5	5
3	3	4	5	4	3	3	4	3
1	1	1	1	1	1	1	1	1
4	5	5	5	5	5	2	3	3
5	5	5	5	5	5	5	5	5
5	5	5	5	5	5	5	5	5
5	5	5	5	5	5	5	5	5
5	5	5	5	5	5	3	3	4
5	5	5	5	5	5	4	4	4

5	5	5	5	5	5	5	5	5
5	5	5	5	5	5	5	5	5
3	3	3	3	3	3	3	3	3
5	5	5	5	3	3	5	5	5
5	5	5	5	5	4	4	4	4
5	5	5	5	5	5	3	3	3
5	5	5	5	5	5	5	5	5
5	5	5	5	5	5	3	5	4
5	4	5	5	4	4	5	4	4
5	5	5	5	5	5	5	5	5
4	4	4	5	4	5	4	4	4
3	3	5	4	5	5	3	3	5
4	4	5	4	5	4	3	4	3
3	3	4	4	4	4	4	4	4
5	5	5	5	5	5	5	5	5
5	5	5	5	5	5	5	5	5
5	5	5	5	5	5	5	5	5
5	5	5	5	5	5	5	5	5
5	5	5	5	5	5	5	5	5
5	5	5	5	5	5	4	4	4
5	5	5	5	5	5	5	5	5
5	5	5	5	5	5	5	5	5
5	5	5	5	5	5	4	4	4
5	4	5	4	3	4	4	4	4
5	5	5	5	5	5	5	5	5
5	5	5	5	5	5	2	1	1
5	5	5	5	5	3	3	3	3
4	5	5	5	5	5	3	3	3
5	5	5	5	5	5	5	5	5
5	5	5	5	5	5	5	5	5
4	4	4	4	4	4	4	4	4
5	5	5	5	5	5	5	5	5
3	3	3	3	3	3	3	3	3
5	5	5	5	5	5	5	5	5
5	5	5	5	5	5	5	5	4
5	5	5	5	5	5	3	3	4
5	5	5	5	5	5	3	3	3
5	4	5	5	5	5	5	5	5
3	5	5	5	5	5	2	2	2
5	5	5	5	5	5	5	5	5
5	5	5	5	5	5	5	5	5
4	4	4	4	4	4	4	4	4
5	5	5	5	5	5	5	5	5
5	5	5	5	5	5	5	5	5
5	5	4	4	5	5	3	4	4
5	5	5	5	4	4	4	4	4
5	4	5	3	4	4	4	2	5
4	4	4	4	4	4	4	4	4
4	4	4	4	4	4	5	5	5
5	4	4	4	4	4	3	3	4
4	4	4	4	4	4	4	4	4
5	5	5	5	5	5	5	5	5
1	1	1	1	1	1	1	1	1
5	5	5	5	5	5	4	4	3

5	5	5	5	5	3	5	5	5
5	5	5	5	5	3	3	3	3
5	2	4	2	4	4	3	4	3
2	5	5	5	5	5	5	4	4
4	3	4	3	3	4	3	4	3
5	5	5	5	5	5	3	4	4
5	5	5	5	3	4	4	4	2
5	5	5	5	5	4	4	5	5
5	5	5	5	5	5	3	3	3
5	5	5	5	5	5	5	5	5
4	3	4	4	4	4	3	4	4
4	4	4	4	4	4	3	4	4
5	4	5	4	3	3	3	4	4
4	4	4	4	4	3	4	4	4
5	5	5	5	5	5	5	5	5
5	5	5	5	5	5	5	5	5
5	5	5	5	5	5	5	5	5
5	5	5	5	5	5	5	5	5
5	5	5	5	5	5	5	5	5
5	5	5	5	5	5	5	5	5
5	5	5	5	5	5	5	5	5
5	5	5	5	5	5	5	5	5
5	5	5	5	5	5	5	5	5
5	5	4	5	3	3	4	3	3
1	1	1	1	1	1	1	1	1
3	5	5	5	5	5	4	4	4
5	5	5	5	5	5	5	5	5
5	5	5	5	5	5	5	5	5
5	3	3	3	3	4	2	3	3
5	5	5	5	5	5	5	5	5
5	4	5	4	5	5	3	4	3
5	5	5	5	5	5	5	5	5
3	3	3	3	3	3	3	3	3
5	5	5	5	5	5	5	5	5
5	4	5	4	5	5	2	3	2
5	5	5	5	5	5	5	5	5
4	4	4	5	5	5	3	2	2
5	5	5	5	5	5	5	5	5
5	5	5	5	5	5	3	3	3
5	5	5	5	5	5	2	4	5
5	4	5	5	3	3	3	3	3
5	5	5	5	5	1	5	5	5
4	3	4	4	3	3	2	3	3
4	4	4	4	4	4	4	4	4
5	5	5	5	5	5	3	3	3
2	3	3	5	5	5	4	4	5
4	4	4	4	4	4	5	4	4
5	5	5	4	5	5	2	4	4
4	5	5	5	5	5	5	5	5
5	5	5	5	5	5	3	3	3
3	3	3	3	3	3	3	3	3
5	5	5	5	5	5	3	4	4
3	3	3	4	4	4	4	4	3
4	4	4	4	4	4	3	3	3

3	5	5	5	5	5	4	5	4
5	5	5	5	5	5	3	4	3
5	4	4	4	4	4	3	3	3
3	4	4	4	3	3	3	3	3
5	5	5	5	5	5	5	5	5
5	5	5	5	5	5	5	5	5
4	4	4	4	4	4	4	4	4
5	5	5	5	5	5	5	5	5
5	4	4	4	5	4	4	4	4
5	5	5	5	5	5	5	5	4
5	5	5	5	5	5	3	3	3
5	5	5	5	5	5	5	5	5
5	5	5	5	5	5	1	1	1
5	4	4	4	4	4	3	3	3
5	5	5	5	5	5	4	4	4
5	5	5	5	5	5	5	5	5
5	5	5	5	5	5	5	5	5
5	5	5	5	5	5	2	2	2
5	5	5	5	5	5	5	5	5
4	4	4	3	3	3	4	4	3
3	4	4	5	3	4	3	5	3
5	5	5	5	5	5	1	1	1
4	5	5	5	4	4	2	2	1
5	5	4	4	3	5	3	3	3
4	5	5	5	5	5	3	4	4
5	5	5	5	5	5	5	5	5
5	5	5	5	5	5	5	5	5
5	5	5	5	5	5	5	5	5
3	5	5	5	5	5	5	5	5
5	5	4	4	5	5	3	4	4
4	5	5	5	5	5	5	3	5
5	5	5	5	5	5	5	5	5
5	5	5	5	5	5	5	5	5
5	5	5	5	5	5	3	3	3
3	2	4	3	4	3	4	3	2
5	5	5	5	5	5	3	5	3
5	5	5	5	5	4	5	3	4
5	5	5	5	5	5	5	5	5
5	5	5	5	5	5	5	5	5
4	4	4	4	4	4	4	4	4
5	4	5	4	4	4	3	3	3
5	5	5	5	5	5	5	5	5
5	5	5	5	5	5	5	5	5
5	5	5	5	5	5	4	4	4
5	5	5	5	5	5	3	5	5
5	5	5	5	5	5	3	3	2
3	4	4	4	4	4	3	3	3
5	5	5	5	5	5	5	5	5
5	5	5	5	5	5	5	5	5
5	5	5	5	5	5	3	3	3
5	5	5	5	5	3	3	3	4
5	5	5	5	5	5	5	3	4
3	3	3	3	3	3	3	3	3
5	5	5	5	5	5	5	5	5

5	5	5	5	5	5	5	5	5
4	5	5	5	5	5	3	3	4
4	4	4	4	4	5	4	4	4
3	5	5	5	5	5	3	3	3
3	3	3	3	3	4	2	2	2
5	5	5	5	4	4	3	4	3
3	3	3	3	3	3	3	3	3
4	5	5	5	5	5	5	5	5
4	4	4	4	3	3	4	4	4
5	4	4	5	3	5	1	3	2
5	4	5	4	5	3	3	5	4
5	5	5	5	4	4	4	4	4
5	5	5	5	5	5	5	5	5
5	5	5	5	5	5	5	5	5
5	5	5	5	5	5	5	5	5
3	4	4	4	4	4	3	3	3
5	5	5	5	5	5	3	2	1
5	5	5	5	5	5	3	5	4
5	4	4	5	5	5	4	4	4
2	4	4	4	4	4	5	5	5
5	5	5	5	5	5	5	5	5
5	4	4	4	4	4	3	3	3
5	4	3	5	5	5	5	5	5
5	5	5	5	5	5	5	5	5
4	5	5	5	5	5	4	4	5
3	4	5	5	5	5	5	5	4
5	5	5	5	5	5	5	5	5
5	5	5	5	5	5	5	5	5
5	5	5	5	5	5	5	5	5
4	4	5	5	5	5	3	3	3
5	5	5	5	5	5	5	5	5
3	3	5	5	5	5	5	5	5
5	5	5	5	5	5	4	4	5
5	5	5	5	5	5	5	5	5
3	4	4	4	4	4	4	4	4
5	5	4	5	4	4	5	5	4
5	5	5	5	5	1	5	5	5
3	4	4	4	3	3	3	3	3
5	5	5	5	5	5	5	5	5
5	5	5	5	5	5	5	5	5
5	5	5	5	5	5	4	4	4
5	5	5	5	5	5	5	5	5
2	4	4	4	4	4	4	4	4
3	2	3	4	3	3	3	3	3
5	5	5	5	5	5	1	1	1
5	5	5	3	3	3	3	3	5
3	5	5	5	5	5	3	3	3
5	4	3	4	4	5	4	4	4
3	3	3	3	3	3	3	3	3
3	5	5	5	5	5	5	5	5
4	4	4	4	4	4	4	4	4
5	5	5	5	5	5	5	5	5
5	5	5	5	5	5	5	5	5
5	5	5	5	5	5	5	5	5

5	5	5	5	5	5	5	4	4
4	4	5	4	4	5	5	5	4
5	5	5	5	4	5	3	3	3
5	5	5	5	5	5	5	5	5
4	5	5	5	5	5	3	2	3
5	5	5	5	5	5	3	3	3
5	5	5	5	5	5	5	5	5
4	5	5	5	5	5	5	5	5
5	5	5	5	5	5	5	5	5
5	5	5	5	5	5	5	5	5
5	5	5	5	5	5	5	5	5
4	4	4	4	4	4	4	4	4
5	5	5	5	5	5	5	5	5
5	5	5	5	5	5	5	5	5
4	4	4	4	4	4	3	2	3
4	4	4	4	5	4	3	3	3
3	3	4	4	3	4	3	3	4
5	5	5	5	5	5	5	5	5
5	5	5	5	5	5	5	5	5
5	5	5	5	5	5	2	4	2
5	5	5	5	5	5	5	5	5
5	5	5	5	5	5	5	5	5
5	5	5	5	5	5	5	5	5
4	5	5	5	5	5	1	1	1
1	5	5	5	3	5	3	5	2
4	5	5	5	5	5	3	2	3
4	5	5	5	4	5	4	5	4
5	3	5	3	5	5	3	3	3
5	5	5	5	5	5	5	5	5
5	5	5	5	5	5	4	4	4
5	5	5	5	5	5	3	4	4
5	5	5	5	5	5	5	2	5
5	5	5	5	5	5	5	5	5
5	4	4	5	5	5	4	4	4
4	4	4	3	4	3	4	4	4
5	5	5	5	4	4	5	5	5
5	5	5	5	5	5	5	5	5
4	4	4	4	5	3	5	5	5
5	5	5	5	5	5	2	2	2
5	4	4	5	4	3	3	3	3
5	5	3	4	1	5	5	5	5
4	4	3	3	3	3	4	4	4
4	4	4	4	4	4	3	3	3
3	3	4	4	4	4	2	3	2
5	5	5	5	5	5	3	5	4
1	3	3	3	3	3	3	3	3
5	4	4	5	4	5	2	3	2
4	4	4	4	4	4	4	4	4
5	5	5	5	5	5	5	5	5
5	5	5	5	5	5	5	5	5
5	5	5	5	5	5	5	5	5
4	3	4	4	4	4	4	3	4
4	4	3	4	3	4	3	4	2
5	5	5	5	5	5	5	5	5

4	5	5	5	5	5	4	4	4
5	5	5	3	3	3	3	5	5
5	4	5	5	5	5	4	5	5
5	5	5	4	3	4	2	3	3
5	4	5	5	5	5	3	5	3
4	2	3	4	5	4	3	3	3
5	5	5	5	5	5	5	5	4
3	5	5	3	5	5	4	5	5
5	4	4	4	3	5	2	2	2
3	5	4	4	5	4	3	3	3
5	5	5	5	5	5	5	5	5
5	5	5	5	5	5	5	4	3
5	5	5	5	5	5	5	5	5
5	5	5	5	5	5	5	5	5
3	3	3	3	3	3	3	3	3
5	5	5	5	5	5	5	5	5
5	5	5	5	5	5	5	5	5
5	5	5	5	5	5	5	5	5
5	5	5	5	5	5	5	5	5
4	4	4	4	4	4	3	3	3
5	5	5	5	5	5	5	5	5
5	5	5	5	5	5	5	5	5
5	5	5	5	5	5	5	5	5
4	5	5	5	5	5	5	5	5
5	5	5	5	5	5	5	5	5
5	5	5	5	5	5	5	5	5
2	4	5	5	3	3	2	2	2
5	4	5	4	4	4	3	3	4
5	5	5	5	5	5	5	5	5

AC4	AR1	AR2	AR3	PN1	PN2	PN3	PN4	PN5
4	5	5	5	5	5	5	5	5
4	4	3	3	3	3	4	3	4
5	5	5	5	5	5	5	3	5
5	5	4	4	5	5	5	5	5
5	5	5	5	5	5	5	5	5
5	5	5	5	5	5	5	5	5
5	5	5	5	5	3	3	2	5
3	3	3	3	3	3	5	3	5
2	2	1	1	2	2	1	3	2
5	5	5	5	5	5	5	5	5
4	2	3	4	2	3	4	2	5
4	4	4	4	4	4	4	4	4
5	5	5	5	5	5	5	5	5
5	5	5	5	5	5	5	5	5
5	5	5	5	5	5	5	5	5
5	5	5	5	5	5	4	5	5
5	5	5	5	5	5	5	5	5
5	5	5	5	5	5	5	5	5
4	4	4	4	4	4	4	4	4
5	5	5	5	5	5	5	5	5
3	5	2	3	5	5	5	5	5
5	5	5	5	5	5	5	5	5
5	5	1	5	5	5	5	5	5
3	4	2	2	5	4	3	3	3
4	3	1	1	4	5	3	2	3
3	3	3	3	3	3	3	3	3
3	4	4	4	4	4	4	4	4
4	5	5	5	5	5	4	3	5
5	5	1	5	5	5	5	1	5
4	4	4	4	4	4	4	4	4
5	5	5	5	5	5	4	4	5
3	4	1	1	3	3	2	2	5
4	3	4	4	3	3	3	4	5
5	5	5	5	5	5	5	5	5
5	5	5	4	3	3	4	3	5
5	5	4	5	5	5	4	4	5
4	3	3	3	4	4	4	3	4
5	5	5	5	5	5	5	5	5
5	5	5	5	5	5	5	5	5
5	5	5	5	5	5	5	5	5
5	5	5	5	5	5	5	5	5
3	3	4	3	3	3	4	4	5
5	5	5	5	4	4	5	4	5
5	5	5	5	5	5	5	5	5
5	5	5	5	5	5	5	5	5
5	4	3	1	5	5	5	5	5
5	3	3	3	4	4	4	4	4
5	5	3	3	5	5	5	5	5
5	5	5	5	5	5	5	5	5
5	5	5	5	5	5	5	5	5
5	5	5	5	5	5	5	5	5
5	5	5	5	5	5	5	5	5
4	2	2	2	5	5	5	5	5

3	4	4	5	5	3	4	3	5
5	5	5	5	5	3	5	3	5
4	4	4	4	4	4	4	4	4
4	5	5	5	4	4	4	5	5
5	5	5	5	5	5	5	5	5
4	4	4	4	4	4	4	3	5
5	5	5	5	5	5	5	5	5
5	5	2	2	2	2	3	2	5
5	5	5	5	5	5	5	5	5
5	5	5	3	5	5	4	5	5
4	4	4	4	4	4	4	4	4
5	4	3	3	4	4	4	4	4
5	5	5	5	5	5	5	5	5
5	5	5	5	5	5	5	5	5
5	5	5	5	5	5	5	5	5
2	2	2	2	3	3	2	4	4
5	5	5	5	5	5	5	5	5
5	5	5	5	5	5	5	5	5
5	5	5	5	5	5	5	5	5
3	2	2	2	3	4	3	2	3
5	4	3	2	5	5	5	5	5
5	5	4	4	5	5	4	5	5
5	4	4	4	5	5	4	5	5
5	5	5	5	5	5	5	5	5
3	3	3	3	3	4	1	1	2
4	5	4	3	4	4	3	4	5
5	5	3	2	5	5	5	5	5
3	5	4	4	5	5	3	3	5
4	3	3	3	4	3	3	3	3
3	4	4	4	4	4	4	4	4
5	5	5	5	5	5	5	5	5
3	3	4	4	4	4	4	4	4
3	4	2	1	5	5	3	5	5
3	2	1	1	4	3	3	4	5
3	3	5	5	3	4	4	5	5
4	4	4	4	5	3	4	4	4
4	4	2	4	4	4	4	4	4
2	4	4	3	3	3	3	3	3
5	5	3	2	5	4	4	4	5
5	4	4	3	5	5	5	5	5
4	5	5	5	4	5	5	5	5
5	5	5	5	5	5	5	5	5
4	4	4	3	4	4	3	3	4
5	5	5	5	5	5	5	5	5
5	5	5	5	5	5	5	5	5
5	5	5	5	5	5	5	5	5
3	3	4	3	3	3	4	3	4
1	1	1	1	1	1	1	1	1
5	5	5	5	5	5	5	4	5
5	5	5	5	5	5	5	5	5
5	5	5	5	5	5	5	5	5
5	5	5	5	5	5	5	5	5
3	3	3	3	3	3	3	3	4
4	5	4	4	4	4	4	4	5

5	5	2	2	5	5	5	5	5
5	5	5	5	5	5	5	5	5
3	3	3	3	3	3	3	3	3
5	5	5	5	5	5	5	5	3
5	4	5	5	5	5	5	5	5
3	3	3	3	4	4	4	4	4
5	5	5	5	5	5	5	4	5
4	4	4	4	4	4	4	4	4
5	5	5	5	5	5	5	4	5
5	5	5	3	5	3	5	3	5
5	4	4	4	5	5	3	3	4
5	5	2	2	3	3	3	3	5
3	4	3	2	4	4	4	4	4
4	4	4	4	4	4	4	4	4
5	5	5	5	5	5	5	5	5
5	5	5	5	5	5	5	5	5
5	5	5	5	5	5	5	5	5
5	5	5	5	5	5	5	5	5
5	5	5	5	5	5	5	5	5
5	5	5	5	5	5	5	5	5
5	5	5	5	5	5	5	5	5
5	5	5	5	5	5	5	5	5
5	5	4	4	5	5	5	4	5
4	4	3	3	4	4	4	3	4
5	5	5	5	5	5	5	5	5
4	5	5	5	5	5	5	5	5
4	3	2	2	3	4	2	2	4
4	3	3	3	5	3	4	3	5
5	5	5	5	5	5	5	5	5
5	5	5	5	5	5	5	5	5
4	4	4	4	4	4	4	4	4
5	5	5	5	5	5	5	5	5
3	3	3	3	3	3	3	3	3
5	5	5	5	5	5	5	5	5
5	3	3	5	5	5	5	2	5
4	5	5	5	4	4	3	3	5
5	5	5	5	5	5	5	5	5
5	5	4	4	5	4	4	4	4
3	4	4	4	5	5	5	5	5
5	5	5	5	5	5	5	5	5
5	5	5	5	5	5	5	5	5
4	4	4	4	4	4	4	4	4
5	5	5	5	5	5	5	5	5
5	5	5	5	5	4	4	5	5
3	3	4	5	4	2	3	3	5
4	4	4	2	4	4	4	3	4
3	4	3	4	5	5	2	4	4
4	4	4	4	4	4	4	4	4
5	4	4	4	4	5	4	4	5
5	3	3	3	3	3	4	2	5
4	4	4	4	4	4	4	3	4
5	5	5	5	5	5	5	5	5
1	1	1	1	1	1	1	1	1
3	4	4	2	5	5	3	3	3

5	2	2	2	3	3	3	3	5
5	5	5	3	3	3	3	2	5
4	5	5	5	3	4	3	4	4
5	3	2	1	3	3	1	4	4
4	4	4	2	4	4	2	2	3
4	5	5	5	5	5	5	3	5
4	4	4	2	4	3	4	2	4
4	5	5	5	5	4	5	2	5
3	4	4	4	4	4	4	4	5
5	5	5	5	5	5	5	5	5
4	4	4	4	5	5	5	5	5
3	4	3	4	3	4	3	3	4
4	5	5	3	4	4	3	4	4
3	3	3	3	4	4	3	3	4
5	5	4	2	1	5	5	1	5
5	5	5	5	5	5	5	5	5
5	5	5	5	5	5	5	5	5
5	5	5	5	5	5	5	5	5
5	5	5	4	4	5	5	5	5
5	5	5	5	5	5	5	5	5
5	5	5	5	5	5	5	5	5
5	5	5	5	5	5	5	5	5
5	5	5	5	5	5	5	5	5
4	4	3	4	3	4	4	5	5
1	1	1	1	1	1	1	1	1
4	4	4	4	4	4	4	4	4
5	5	5	5	5	5	5	5	5
5	5	5	5	5	5	5	5	5
3	3	3	3	3	3	1	3	3
5	5	5	5	5	5	4	4	5
3	3	3	3	3	3	2	1	3
5	5	5	5	5	5	5	5	5
3	3	3	3	3	3	3	3	3
5	5	5	5	5	5	5	5	5
4	5	5	4	5	5	5	4	5
5	5	5	5	5	5	5	5	5
2	4	4	4	4	4	4	4	4
5	5	5	1	5	5	5	5	5
3	4	4	3	4	4	3	3	3
5	5	4	4	5	5	4	3	5
5	4	4	2	4	4	4	3	5
5	5	5	5	5	3	2	1	5
3	3	3	2	3	3	3	2	4
4	4	4	4	4	4	4	4	4
3	4	4	4	3	3	3	3	3
4	2	2	2	5	5	3	3	3
4	5	5	5	5	5	4	4	4
5	5	4	4	5	5	4	4	5
5	5	5	5	5	5	5	5	5
3	3	3	3	3	3	3	3	3
3	3	3	3	3	3	3	3	3
4	5	5	5	5	5	5	5	5
4	4	3	3	4	3	4	3	4
3	4	3	2	4	4	4	4	4

5	4	5	4	5	4	5	4	5
4	4	3	3	4	4	4	2	5
4	3	3	3	4	4	4	4	4
5	4	3	2	5	5	5	5	5
5	5	5	5	5	5	5	5	5
5	5	5	5	5	5	5	5	5
4	4	4	4	4	4	4	4	4
5	5	5	5	5	5	5	5	5
4	4	4	2	4	4	3	3	4
5	5	5	5	5	5	3	4	5
5	4	4	3	4	4	4	4	5
5	5	3	5	5	5	3	4	5
5	5	3	3	5	5	5	5	5
4	4	4	3	4	4	4	4	4
5	5	3	3	5	5	5	3	5
5	5	5	5	5	5	5	5	5
5	5	5	5	5	5	5	5	5
5	5	5	5	5	5	4	5	5
5	5	5	5	5	5	5	5	5
4	4	3	4	4	4	3	4	3
4	4	5	5	4	4	4	4	5
5	5	5	5	5	5	5	5	5
4	4	3	3	4	5	3	3	4
3	2	2	3	3	5	4	4	4
3	5	5	3	5	5	5	5	4
5	5	5	5	5	5	5	5	5
5	5	5	5	5	5	5	5	5
5	5	5	5	5	5	5	5	5
5	5	5	5	5	5	5	5	5
4	5	5	3	5	5	5	3	5
5	5	5	5	5	5	5	5	5
5	5	5	5	5	5	5	5	5
5	5	5	5	5	5	5	5	5
5	5	5	5	5	5	5	3	5
4	5	3	3	4	4	4	5	5
5	5	5	4	5	5	4	5	5
4	4	3	4	3	4	3	2	3
5	5	4	4	5	5	5	4	4
5	5	5	5	5	5	5	5	5
4	4	4	4	4	4	4	4	4
3	3	3	4	4	5	4	4	2
5	5	5	5	5	5	5	5	5
5	5	5	5	5	5	5	5	5
5	5	4	4	5	5	5	5	5
5	5	5	5	5	4	5	4	5
5	5	5	4	5	5	5	5	5
3	3	3	3	3	3	3	3	3
5	5	5	5	5	5	5	5	5
5	5	5	5	5	5	5	5	5
5	5	5	3	5	5	5	5	5
5	3	4	3	4	4	3	3	4
5	5	5	5	5	5	5	5	5
3	3	3	3	3	3	3	3	3
5	5	5	5	5	5	5	5	5

5	5	5	5	5	5	5	5	5
4	4	4	4	4	4	4	4	4
4	4	5	5	5	5	4	3	3
5	5	5	5	5	5	5	5	5
2	2	2	2	2	2	2	3	3
5	5	4	4	5	5	5	3	4
3	3	3	3	3	3	3	3	3
5	5	5	5	5	5	5	5	5
4	3	3	3	3	3	4	3	4
5	4	5	4	2	5	2	2	5
5	5	4	2	4	4	4	4	4
4	4	4	4	5	5	5	5	5
5	5	5	5	5	5	5	5	5
5	5	5	5	5	5	5	5	5
5	5	5	5	5	5	5	5	5
3	3	3	3	3	3	3	3	3
5	5	3	2	5	5	5	5	5
5	4	3	3	5	5	4	5	5
5	5	5	5	5	5	5	5	5
5	4	5	4	5	4	5	4	5
5	5	5	5	5	5	5	5	5
3	3	5	4	3	3	3	3	5
5	5	5	5	5	5	5	5	5
5	4	5	5	5	5	5	5	5
5	5	4	5	5	5	5	5	5
5	4	3	3	5	5	5	5	5
5	5	5	5	5	5	5	5	5
5	5	5	5	5	5	5	5	5
5	5	5	5	5	5	5	5	5
3	4	3	3	4	3	3	2	5
5	5	5	5	5	5	5	5	5
5	5	5	5	5	5	5	5	5
5	5	4	3	5	5	5	5	5
5	5	5	5	5	5	5	5	5
4	4	4	4	4	4	4	4	4
4	4	4	4	5	5	5	5	5
5	5	5	5	5	5	5	5	5
4	4	3	2	3	3	3	3	4
5	5	5	5	5	5	5	5	5
5	5	5	5	5	5	5	5	5
5	5	5	5	5	5	5	5	5
5	5	5	5	5	5	5	5	5
4	4	4	4	4	4	4	4	4
3	3	3	3	3	3	3	2	3
2	3	3	2	3	3	5	3	5
5	5	5	5	5	5	5	5	5
3	3	3	3	5	5	5	4	5
4	4	5	1	4	3	3	3	4
3	3	3	3	3	3	3	3	3
5	5	5	5	5	5	5	5	5
4	4	5	5	5	5	5	5	5
5	5	5	5	5	5	5	5	5
5	5	5	5	5	5	5	5	5
5	5	5	5	5	5	5	5	5

4	5	5	5	5	5	5	5	5
5	4	5	4	5	4	5	4	5
5	5	3	3	5	4	3	3	5
5	5	5	5	5	5	5	5	5
4	4	4	4	4	4	4	4	4
5	5	5	5	5	5	5	5	5
5	5	5	4	4	3	4	1	4
5	5	5	5	5	5	5	5	5
5	5	5	5	5	5	5	5	5
5	5	5	5	5	5	5	5	5
5	5	5	5	5	5	5	5	5
4	4	4	4	4	4	4	4	4
5	5	5	5	5	5	5	5	5
5	5	5	5	5	5	5	5	5
4	4	4	3	3	3	3	3	3
4	5	3	2	4	4	4	4	5
4	3	4	4	3	3	3	3	3
5	5	5	5	5	5	5	5	5
5	5	5	5	5	5	5	5	5
5	5	5	5	5	5	5	5	5
5	5	5	5	5	5	5	5	5
5	5	5	5	5	5	5	5	5
5	5	3	4	5	5	5	3	5
1	5	1	1	4	5	2	1	1
5	5	5	5	5	5	4	3	4
5	5	5	5	5	5	3	3	5
5	5	4	3	5	5	4	4	5
3	3	3	1	1	4	1	4	3
5	5	5	5	5	5	5	5	5
5	5	5	5	5	5	5	5	5
4	4	4	4	4	4	4	5	5
5	5	5	5	5	5	5	5	5
5	5	5	5	5	5	5	5	5
5	4	5	4	5	5	5	5	5
4	2	2	2	2	2	2	2	2
5	5	5	5	5	5	4	4	5
5	5	5	5	5	5	5	5	5
5	5	5	5	5	5	5	2	4
4	5	5	4	5	5	5	3	5
3	5	4	4	2	3	3	5	5
5	5	4	4	5	5	5	5	5
4	4	4	4	3	3	3	3	3
3	3	3	3	3	3	3	3	1
3	4	4	4	3	3	3	2	5
5	5	5	2	3	4	5	3	5
3	3	3	3	3	3	3	3	3
4	3	2	3	4	4	4	3	4
4	4	4	4	4	4	4	4	4
5	5	5	5	5	5	5	5	5
5	5	5	5	5	5	5	5	5
5	5	5	5	5	5	5	5	5
3	3	3	3	4	4	4	4	4
4	4	3	4	4	2	4	5	4
5	5	5	5	5	5	5	5	5

4	4	4	4	5	5	5	5	5
5	5	5	5	5	5	5	3	5
5	4	4	3	4	4	4	3	5
4	3	3	3	4	3	3	4	4
4	5	3	1	4	4	3	2	4
5	1	1	1	1	1	1	1	3
3	5	5	3	3	5	3	3	5
5	5	5	5	5	5	5	5	5
3	3	3	1	2	2	2	2	3
4	3	3	3	3	3	3	3	3
5	5	5	5	5	5	5	5	5
5	4	5	5	5	5	5	4	4
5	5	5	5	5	5	5	5	5
5	5	5	5	5	5	5	5	5
3	3	4	4	4	3	4	4	4
5	5	5	5	5	5	5	5	5
5	5	5	5	5	5	5	5	5
5	5	5	5	5	5	5	5	5
5	5	5	5	5	5	5	5	5
3	4	4	4	4	4	4	3	4
5	5	5	5	5	5	5	5	5
5	5	5	5	5	5	5	5	5
5	5	5	5	5	5	5	5	5
5	4	4	5	4	4	4	4	4
5	5	5	4	5	4	4	3	5
5	5	5	5	5	5	5	5	5
3	5	2	1	2	2	5	1	5
5	5	3	4	5	5	5	5	5
5	5	5	5	5	5	5	3	5

Gender	Age	Educatior	Educatior	Frequence	Frequence	AV	BV	CV
1	3	2	2	7	5	15	20	15
1	3	2	2	7	4	11	19	12
1	2	2	3	7	4	14	20	15
1	2	2	3	7	6	13	20	9
2	2	2	1	7	4	12	20	15
2	2	2	1	7	7	15	20	15
2	2	2	1	7	7	14	20	9
2	3	2	1	7	4	9	20	9
2	2	2	1	7	5	15	19	15
2	2	2	1	7	5	15	20	15
1	2	2	1	7	4	9	12	11
1	4	2	1	7	4	12	16	12
1	3	2	1	7	4	13	20	15
1	4	2	2	7	4	15	20	15
2	4	2	2	7	7	15	20	15
2	3	2	2	7	6	13	20	15
2	3	2	2	7	4	15	20	15
2	4	2	2	7	4	14	20	11
1	4	2	2	7	7	12	16	12
1	4	2	2	7	7	3	20	15
1	3	2	2	7	4	11	20	15
1	4	3	2	7	7	15	20	15
1	4	2	2	7	7	15	20	15
1	4	2	2	7	7	15	20	15
1	4	2	2	7	7	13	20	14
1	4	2	3	7	7	8	12	13
1	4	2	3	7	4	11	20	9
1	5	2	3	7	7	14	20	9
1	4	2	3	7	3	3	20	15
1	4	2	3	7	1	12	16	12
1	8	2	3	7	7	13	20	13
2	5	2	3	7	6	12	19	14
2	5	2	3	7	7	12	20	12
2	4	2	3	7	7	15	20	15
2	4	2	3	7	4	15	20	15
2	5	2	3	3	7	15	20	15
2	4	2	3	3	3	14	20	15
2	4	2	3	7	1	15	20	15
2	8	2	3	7	7	15	20	15
2	4	2	3	7	7	15	20	15
2	4	2	3	7	4	15	20	15
1	4	2	3	7	3	13	17	12
1	5	2	3	7	3	15	16	14
1	4	2	3	7	3	15	20	15
2	6	2	4	7	4	15	20	15
2	4	2	4	7	5	15	20	15
2	6	2	4	7	7	13	20	15
2	6	2	4	7	4	13	20	15
2	2	2	4	7	2	11	20	15
2	5	2	4	7	4	15	20	15
2	6	2	4	7	5	13	20	15
1	9	3	5	7	7	15	20	15
1	2	2	2	6	4	15	20	15

2	4	2	1	6	3	15	20	15
2	2	2	1	6	3	11	20	12
1	3	2	1	3	4	12	16	12
1	3	2	1	3	4	11	19	13
2	4	2	2	3	4	15	20	15
2	4	2	2	3	3	12	19	12
2	5	2	2	3	3	15	20	15
2	4	2	2	3	4	15	20	15
1	3	2	2	3	3	15	20	15
1	4	2	3	3	3	15	20	15
1	4	2	3	3	5	13	12	12
1	4	2	3	3	4	10	20	12
2	4	2	3	3	3	15	20	15
2	4	2	3	6	5	15	20	15
2	4	2	3	6	4	10	20	15
1	5	2	3	6	4	10	20	12
1	5	2	3	6	4	15	20	15
2	6	2	4	6	1	15	20	15
2	5	2	4	6	3	15	20	15
2	5	2	4	6	4	15	20	15
1	5	2	4	6	4	11	20	13
1	9	3	5	6	4	15	20	12
1	9	3	5	6	4	14	20	14
1	2	2	2	5	5	9	20	15
1	3	2	2	5	4	14	17	12
1	3	2	3	5	4	15	19	15
1	2	2	3	5	4	14	20	15
1	2	2	3	5	4	15	19	15
2	3	2	3	5	4	9	20	14
2	2	2	3	5	5	12	16	12
2	3	2	1	5	4	15	20	15
2	2	2	1	5	2	13	20	12
2	2	2	1	5	3	15	18	15
2	2	2	1	5	2	13	20	12
1	2	2	1	5	3	7	20	9
1	5	2	3	5	3	13	16	12
1	4	2	3	5	3	10	16	12
1	6	2	3	5	4	3	20	11
1	4	2	3	5	3	14	20	15
2	4	1	3	5	4	14	20	15
2	5	2	3	5	3	15	18	15
2	4	2	3	5	4	15	20	15
2	4	2	3	5	3	12	16	12
2	4	2	3	5	3	15	20	15
2	5	2	3	5	3	15	20	15
2	4	2	3	5	3	15	20	15
2	6	2	3	5	4	9	20	13
2	4	2	3	5	3	6	20	3
1	5	2	3	5	5	12	19	15
1	5	2	3	5	5	15	20	15
2	6	2	4	5	1	15	20	15
2	6	2	4	5	4	15	20	15
2	5	2	4	5	3	11	20	15
2	6	2	4	5	4	14	20	15

1	5	2	4	5	3	15	20	15
1	4	2	2	4	3	15	20	15
1	2	2	2	4	3	9	12	9
1	1	2	2	4	3	11	20	13
1	2	2	2	4	4	15	20	15
2	2	2	1	4	4	15	20	15
2	3	2	1	4	6	13	20	15
2	3	2	1	4	4	15	16	15
2	4	2	1	4	2	13	20	14
2	2	2	1	4	2	15	20	15
2	3	2	1	4	4	12	20	13
2	2	2	1	4	3	10	20	14
1	4	2	1	4	4	12	16	14
1	4	2	1	4	1	11	16	12
1	3	2	1	4	3	15	20	15
2	2	2	2	4	4	15	20	15
1	3	2	2	4	2	15	20	15
1	3	2	2	4	3	15	20	15
1	3	2	2	4	2	12	20	15
1	3	2	2	4	3	15	20	15
1	5	2	2	4	3	12	20	15
2	3	2	2	4	3	15	20	15
2	4	2	2	4	3	13	20	15
2	4	2	2	4	3	15	20	12
2	2	2	2	4	2	15	20	15
2	4	2	2	4	4	10	20	15
2	3	2	2	4	2	12	20	15
2	4	2	2	4	3	11	20	15
2	4	2	2	4	5	15	20	15
2	4	2	2	4	3	15	20	15
2	3	2	2	4	2	12	16	12
2	2	2	2	4	3	15	20	15
2	3	2	2	4	4	9	12	9
2	4	2	2	4	3	15	20	15
2	5	2	2	4	5	13	20	15
2	4	2	2	4	4	15	20	15
2	2	2	2	4	4	15	20	15
2	3	2	2	4	2	13	20	15
2	3	2	2	4	3	6	20	15
2	3	2	2	4	2	15	20	15
2	3	2	2	4	3	15	20	15
2	5	2	2	4	3	12	16	12
1	5	2	2	4	4	15	19	15
1	5	2	2	4	3	15	20	15
1	6	2	2	4	3	11	14	13
1	4	2	2	4	2	14	19	14
1	4	2	2	4	4	13	16	12
1	3	2	2	4	1	12	16	12
1	3	2	2	4	3	10	20	12
1	5	2	2	4	4	14	20	12
1	6	2	3	4	4	11	12	12
1	5	2	3	4	2	15	20	15
1	4	2	3	4	3	3	4	3
1	4	2	3	4	3	12	20	15

1	4	2	3	4	1	15	20	15
1	4	2	3	4	2	15	20	15
1	4	2	3	4	3	11	18	10
1	4	2	3	4	2	7	20	15
1	5	2	3	4	4	14	16	10
1	5	2	3	4	2	10	20	15
1	4	2	3	4	2	11	20	13
1	5	2	3	4	3	11	19	15
1	4	2	3	4	1	13	20	15
2	5	2	3	4	1	15	20	15
2	5	2	3	4	1	8	20	12
2	5	2	3	4	1	13	20	12
2	5	2	3	4	4	13	20	12
2	6	2	3	4	3	12	14	12
2	5	2	3	4	3	15	20	15
2	6	2	3	4	4	15	20	15
2	5	2	3	4	2	15	20	15
2	4	2	3	4	3	15	20	15
2	4	2	3	4	3	15	20	15
2	4	2	3	4	1	15	20	15
2	4	2	3	4	2	15	20	15
2	4	2	3	4	3	15	20	15
2	4	2	3	4	2	15	20	15
2	5	2	3	4	4	13	19	12
2	5	2	3	4	2	3	4	3
2	4	2	3	4	2	8	20	15
2	5	2	3	4	3	15	20	15
2	4	2	3	4	1	15	20	15
2	4	2	3	4	3	10	17	9
1	5	3	3	4	4	10	19	15
1	4	2	3	4	2	11	19	14
1	4	2	3	4	7	12	20	15
1	5	3	3	4	4	9	20	9
2	1	3	4	4	2	15	20	15
2	7	2	4	4	2	11	20	14
2	5	2	4	4	2	15	20	15
2	7	2	4	4	1	10	20	14
2	6	2	4	4	2	15	20	15
2	6	2	4	4	3	15	20	15
2	6	2	4	4	3	13	16	15
2	6	2	4	4	2	15	18	13
2	4	2	2	3	3	15	20	15
2	4	2	2	3	3	10	19	11
2	3	2	2	3	4	12	16	12
1	4	2	3	3	4	12	18	15
1	4	2	3	3	3	4	20	13
1	4	2	3	3	3	12	15	12
2	4	2	2	3	1	15	20	14
1	4	2	2	3	1	14	20	15
1	5	2	2	3	1	15	20	15
1	5	1	2	3	2	9	12	9
1	2	2	2	3	2	15	20	15
1	2	2	2	3	1	9	13	11
1	2	2	2	3	3	10	20	12

1	2	2	3	3	3	11	20	15
1	2	2	3	3	4	14	20	15
1	2	2	3	3	3	15	20	12
1	4	2	3	3	2	10	20	11
2	2	2	3	3	3	15	20	15
2	2	2	3	3	1	15	20	15
2	2	2	3	3	3	12	16	12
2	3	2	1	3	3	15	20	15
2	2	2	1	3	3	15	16	13
2	3	2	1	3	3	14	20	15
2	3	2	1	3	3	13	18	15
2	2	2	1	3	4	15	20	15
2	1	2	1	3	3	15	20	15
2	3	2	1	3	3	15	19	12
1	2	2	1	3	4	15	20	15
1	4	2	1	3	3	15	20	15
1	2	2	1	3	3	15	20	15
1	3	2	1	3	3	15	19	15
2	4	2	2	3	4	15	20	15
1	2	2	2	3	1	11	15	10
1	4	2	2	3	2	11	19	12
1	3	2	2	3	3	15	20	15
1	2	2	3	3	2	10	18	14
1	2	2	3	3	2	12	18	11
2	2	2	3	3	2	11	20	15
2	2	2	3	3	2	15	20	15
2	2	2	3	3	3	15	20	15
2	2	2	1	3	2	15	20	15
2	2	2	1	3	2	8	20	15
2	3	2	1	3	3	14	19	13
2	2	1	1	3	1	7	20	15
2	3	2	1	3	2	15	20	15
2	3	2	1	3	3	15	20	15
2	3	2	1	3	2	11	20	15
2	3	2	1	3	1	8	20	11
2	3	2	1	3	2	14	20	15
1	2	2	1	3	1	14	20	15
1	2	2	1	3	2	15	17	15
1	4	2	2	3	5	15	20	15
1	4	2	2	3	1	12	16	12
1	4	2	2	3	2	11	19	13
2	3	2	2	3	3	15	20	15
2	3	2	2	3	1	15	20	15
2	4	2	2	3	1	14	20	15
2	4	2	2	3	1	14	20	15
2	4	2	2	3	1	11	20	15
2	3	2	2	3	2	9	20	12
2	3	2	2	3	3	15	20	15
2	4	2	2	3	2	15	20	15
2	5	2	2	3	2	11	20	15
2	4	2	2	3	5	15	20	15
2	4	2	2	3	1	14	20	15
2	4	2	2	3	2	9	12	9
1	4	3	2	3	3	15	20	15

1	4	2	3	3	2	15	20	15
1	4	2	3	3	4	12	20	15
1	4	2	3	3	1	12	20	12
1	4	2	3	3	2	13	20	15
1	4	2	3	3	3	9	16	9
1	5	2	3	3	3	15	19	14
2	4	2	3	3	2	9	12	9
2	4	2	3	3	2	10	20	15
2	4	2	3	3	3	12	20	11
2	5	2	3	3	1	10	20	12
2	5	2	3	3	1	15	19	14
2	4	2	3	3	2	14	16	14
2	4	2	3	3	4	15	18	15
2	4	2	3	3	1	15	20	15
2	4	2	3	3	2	15	20	15
2	4	2	3	3	3	10	16	12
2	5	2	3	3	3	15	20	15
2	4	2	3	3	2	15	20	15
1	5	2	3	3	1	13	20	14
1	4	2	2	2	1	6	20	12
1	4	2	2	2	1	15	20	15
1	5	2	2	2	1	11	20	12
1	2	2	2	2	1	13	20	13
1	3	2	2	2	2	15	20	15
1	3	2	2	2	1	13	20	15
1	2	2	3	2	1	10	20	15
2	4	2	3	2	1	15	20	15
2	3	2	1	2	1	15	20	15
2	1	2	1	2	1	15	20	15
2	2	2	1	2	1	12	20	15
2	2	2	1	2	1	15	20	15
2	1	2	1	2	1	13	20	15
2	3	2	1	2	1	12	20	15
2	3	2	1	2	1	15	20	15
2	4	2	1	2	1	9	16	12
2	3	2	1	2	1	13	20	13
2	2	2	1	2	2	15	20	15
2	3	2	1	2	1	9	17	11
2	2	2	1	2	1	15	20	15
2	3	2	1	2	1	15	20	15
2	4	2	1	2	1	15	20	15
2	2	2	1	2	1	15	20	15
2	2	2	1	2	1	7	20	12
2	2	2	1	2	1	12	19	10
2	2	2	1	2	1	15	20	15
2	3	2	1	2	1	11	20	11
2	3	2	1	2	1	11	20	15
2	2	2	1	2	5	15	20	11
2	2	2	1	2	1	9	12	9
1	3	2	1	2	1	11	20	15
1	1	2	1	2	1	12	18	12
1	2	2	1	2	1	15	20	15
1	2	2	1	2	1	15	20	15
2	4	2	2	2	1	15	20	15

1	4	2	2	2	1	14	19	15
2	2	2	2	2	1	13	19	13
2	4	2	2	2	4	13	20	14
2	5	2	2	2	1	15	20	15
2	4	2	2	2	1	10	19	15
2	4	2	2	2	1	15	20	15
2	4	2	2	2	1	13	20	15
2	3	2	2	2	1	12	20	15
2	4	2	2	2	1	15	20	15
2	3	2	2	2	1	15	20	15
2	3	2	2	2	1	15	20	15
2	4	2	2	2	1	12	16	12
2	4	2	2	2	1	15	20	15
1	2	2	2	2	1	15	20	15
1	2	2	2	2	2	13	15	12
1	3	2	3	2	2	11	20	13
1	3	2	3	2	1	13	13	11
2	3	2	3	2	2	15	20	15
2	4	2	3	2	2	15	20	15
2	3	2	1	2	1	15	20	15
2	4	2	1	2	2	15	20	15
2	2	2	1	2	1	15	20	15
2	2	2	1	2	2	15	20	15
2	3	2	1	2	2	14	20	15
2	3	2	1	2	1	11	14	13
2	4	2	1	2	2	10	20	15
2	3	2	1	2	2	13	20	14
1	2	2	1	2	1	10	18	13
1	3	2	2	2	2	15	20	15
1	4	2	2	2	2	15	20	15
1	3	2	2	2	2	15	20	15
2	4	2	2	2	2	15	20	15
2	3	2	2	2	2	15	20	15
2	4	2	2	2	2	15	20	14
2	4	2	2	2	1	12	18	11
2	3	2	2	2	2	13	20	14
2	4	2	2	2	2	15	20	15
2	3	2	2	2	2	12	20	13
1	5	2	3	2	1	15	20	15
1	5	2	3	2	1	11	20	13
1	7	2	3	2	2	14	20	8
1	5	2	3	2	1	10	16	9
1	5	2	3	2	1	12	20	12
2	4	2	3	2	1	7	18	12
2	5	2	3	2	1	15	20	15
2	5	2	3	2	1	5	16	9
2	7	2	3	2	2	9	20	13
2	5	2	3	2	1	12	16	12
2	5	2	3	2	1	15	20	15
2	7	2	4	2	2	15	20	15
1	3	2	1	1	4	14	19	15
1	3	2	2	1	3	11	14	12
1	2	2	2	1	3	9	16	10
1	5	2	3	1	1	15	20	15

1	5	2	3	1	1	12	20	15
1	5	2	3	1	1	13	20	11
1	5	2	3	1	1	12	20	15
1	5	2	3	1	1	13	17	12
1	5	2	3	1	4	15	18	15
1	3	2	3	1	1	9	17	12
2	5	2	3	1	1	12	20	15
2	5	2	3	1	1	8	20	13
2	4	2	3	1	5	15	18	11
2	4	2	3	1	1	9	20	13
2	5	2	3	1	1	15	20	15
2	5	2	3	1	1	13	20	15
2	5	2	3	1	1	15	20	15
2	5	2	3	1	1	15	20	15
2	5	2	3	1	1	11	16	9
2	5	2	3	1	4	13	20	15
2	3	2	3	1	1	15	20	15
1	4	2	3	1	1	15	20	15
1	5	2	3	1	2	15	20	15
1	4	2	3	1	1	11	20	12
1	4	2	3	1	1	15	20	15
2	7	2	4	1	1	15	20	15
2	7	2	4	1	1	15	20	15
2	5	2	4	1	4	13	20	15
2	6	2	4	1	1	14	20	15
2	5	2	4	1	1	15	20	15
2	5	2	4	1	1	6	18	13
2	6	2	4	1	1	15	20	13
2	3	2	5	1	2	15	20	15

GPA	EA	AC	AR	PN	SN	PBC	Intention
25	15	7	15	20	15	15	20
20	12	11	10	13	10	12	16
20	15	15	15	18	4	8	13
25	15	11	13	20	9	10	20
20	12	15	15	20	11	15	15
25	15	15	15	20	8	5	15
25	15	6	15	13	15	15	20
15	15	9	9	14	11	11	12
20	15	6	4	8	9	11	9
25	15	15	15	20	15	15	20
15	9	9	9	11	9	9	12
20	12	12	12	16	12	12	16
25	15	15	15	20	15	15	20
25	15	10	15	20	11	14	19
25	15	15	15	20	15	15	20
17	13	15	15	19	9	14	15
25	15	15	15	20	15	15	20
25	12	15	15	20	15	15	20
20	12	12	12	16	12	12	16
25	15	15	15	20	15	15	20
25	9	7	10	20	11	11	18
25	15	15	15	20	15	15	20
25	15	5	11	20	15	15	20
25	15	6	8	15	9	15	20
20	14	8	5	14	6	13	20
15	12	9	9	12	9	9	12
25	15	9	12	16	15	15	16
15	9	13	15	17	9	9	12
25	15	14	11	16	15	15	20
20	15	12	12	16	12	12	16
21	15	12	15	18	10	11	18
25	12	8	6	10	9	8	11
25	15	11	11	13	15	15	20
25	15	15	15	20	15	15	20
24	15	10	14	13	12	11	17
22	12	15	14	18	12	12	16
18	9	10	9	15	9	13	12
19	12	15	15	20	11	13	16
25	15	15	15	20	15	15	20
25	15	15	15	20	15	15	20
25	15	15	15	20	15	15	20
13	9	9	10	14	4	6	11
22	10	15	15	17	15	12	18
25	15	15	15	20	15	15	20
25	15	15	15	20	15	15	20
25	15	8	8	20	15	15	20
25	15	12	9	16	5	11	12
25	12	15	11	20	9	11	16
25	15	15	15	20	3	13	14
25	15	15	15	20	15	15	20
25	15	15	15	20	9	13	20
25	15	15	15	20	15	15	20
25	15	13	6	20	15	15	20

20	15	11	13	15	12	13	14
25	15	9	15	16	7	15	19
20	10	15	12	16	12	10	16
20	12	11	15	17	9	13	16
25	15	15	15	20	15	15	20
21	12	13	12	15	12	12	15
25	15	13	15	20	12	9	19
25	15	9	9	9	3	13	20
25	15	15	15	20	15	15	20
24	15	15	13	19	13	13	16
17	9	12	12	16	9	10	12
20	12	12	10	16	12	9	16
25	15	15	15	20	15	15	20
25	15	15	15	20	15	13	20
25	15	11	15	20	15	15	19
25	15	6	6	12	10	15	17
25	15	15	15	20	15	15	20
25	15	15	15	20	15	15	20
24	15	15	15	20	15	15	16
25	15	9	6	12	15	15	20
23	15	11	9	20	8	12	20
25	15	15	13	19	14	14	20
25	15	12	12	19	15	14	20
25	15	15	15	20	9	9	20
20	9	10	9	9	8	14	14
24	15	12	12	15	11	13	19
24	15	12	10	20	11	12	20
20	15	8	13	16	11	8	15
25	15	8	9	13	4	11	20
20	12	9	12	16	12	12	15
25	15	15	15	20	15	15	20
14	9	9	11	16	3	10	11
25	15	9	7	18	9	10	20
20	15	10	4	14	11	9	17
25	15	9	13	16	15	15	20
21	12	11	12	16	12	12	16
20	12	11	10	16	12	12	16
20	15	6	11	12	7	6	20
25	15	12	10	17	15	6	20
25	15	11	11	20	14	14	19
19	12	9	15	19	8	13	15
25	15	15	15	20	15	15	20
20	12	10	11	14	9	10	16
25	15	15	15	20	15	15	20
25	15	15	15	20	15	15	20
25	15	15	15	20	15	15	20
15	9	10	10	13	9	8	12
25	15	3	3	4	15	15	20
20	15	8	15	19	7	11	13
25	15	15	15	20	15	15	20
25	15	15	15	20	15	15	20
24	9	15	15	20	9	12	16
17	9	10	9	12	3	6	9
24	12	12	13	16	9	11	18

25	15	15	9	20	15	15	20
25	15	15	15	20	15	15	20
15	9	9	9	12	8	8	12
25	15	15	15	20	14	15	20
25	15	12	14	20	15	15	20
15	9	9	9	16	9	9	12
25	15	15	15	19	15	15	20
18	15	12	12	16	6	11	12
25	15	13	15	19	15	15	20
25	15	15	13	16	9	10	15
20	12	12	12	16	9	13	17
25	15	11	9	12	15	15	20
15	12	10	9	16	9	10	16
20	12	12	12	16	12	12	16
25	15	15	15	20	15	15	20
25	15	15	15	20	15	15	20
25	15	15	15	20	15	15	20
25	15	15	15	20	15	15	20
25	15	15	15	20	15	13	20
25	15	12	15	20	15	15	20
25	15	15	15	20	15	15	20
25	15	15	15	20	12	12	20
25	14	12	13	19	14	14	20
22	15	12	10	15	6	10	16
25	15	15	15	20	15	15	20
25	15	4	15	20	12	13	20
19	8	9	7	11	6	10	16
25	15	9	9	15	12	13	16
25	15	15	15	20	15	15	20
25	15	15	15	20	15	15	20
20	12	12	12	16	12	12	16
25	15	15	15	20	15	15	20
15	9	9	9	12	9	9	12
25	15	15	15	20	15	15	20
25	15	14	11	17	15	15	20
17	9	10	15	14	6	13	13
25	15	9	15	20	12	14	20
24	15	15	13	17	9	10	13
25	12	6	12	20	7	9	20
25	15	15	15	20	15	15	20
25	15	15	15	20	15	15	20
20	15	12	12	16	12	15	16
25	15	15	15	20	15	15	20
21	15	15	15	18	9	11	20
19	11	11	12	12	8	9	17
20	15	12	10	15	11	13	14
21	14	11	11	16	11	12	15
20	12	12	12	16	12	12	16
25	12	15	12	17	9	9	16
19	12	10	9	12	8	9	15
15	9	12	12	15	9	9	12
19	15	15	15	20	9	9	16
5	3	3	3	4	3	3	4
20	15	11	10	16	12	12	16

20	12	15	6	12	3	11	16
25	12	9	13	11	12	10	20
21	12	10	15	14	12	9	12
20	15	13	6	11	6	9	12
16	9	10	10	12	9	11	12
21	15	11	15	18	10	7	9
25	15	10	10	13	12	7	18
23	13	14	15	16	11	12	16
13	10	9	12	16	8	12	12
25	15	15	15	20	15	15	20
20	12	11	12	20	12	14	15
16	8	11	11	13	8	10	12
16	10	11	13	15	6	8	10
23	11	12	9	14	11	8	18
25	15	15	11	12	10	13	20
25	15	15	15	20	15	15	20
25	15	15	15	20	15	15	20
25	15	15	15	20	15	15	20
15	15	15	14	19	9	13	19
25	15	15	15	20	15	15	20
25	14	15	15	20	15	15	20
25	15	15	15	20	15	15	20
25	15	15	15	20	15	15	20
17	15	10	11	16	9	11	12
5	3	3	3	4	7	5	4
15	12	12	12	16	9	10	12
25	15	15	15	20	15	15	20
25	15	15	15	20	9	15	20
19	11	8	9	10	9	6	12
23	13	15	15	18	13	12	20
25	15	10	9	9	9	10	14
25	15	15	15	20	15	15	20
15	9	9	9	12	9	9	12
25	15	15	15	20	15	15	20
25	15	7	14	19	15	15	20
25	15	15	15	20	15	15	20
20	12	7	12	16	12	12	16
22	13	15	11	20	6	11	15
18	9	9	11	14	3	4	8
21	15	11	13	17	7	7	20
18	12	9	10	15	9	14	12
22	11	15	15	11	4	11	15
19	12	8	8	11	7	10	15
20	12	12	12	16	12	12	16
21	15	9	12	12	4	5	16
25	15	13	6	16	9	15	20
17	15	13	15	18	12	11	15
25	12	10	13	18	9	14	18
25	15	15	15	20	14	12	20
19	9	9	9	12	3	7	4
15	9	9	9	12	9	9	12
25	15	11	15	20	9	14	20
18	12	11	10	14	9	14	12
23	12	9	9	16	11	13	15

18	12	13	13	18	9	11	16
23	12	10	10	14	10	10	16
22	12	9	9	16	9	9	15
25	15	9	9	20	10	13	18
25	15	15	15	20	15	15	20
25	15	15	15	20	15	15	20
20	12	12	12	16	12	12	16
25	15	15	15	20	15	15	20
19	12	12	10	14	10	12	14
25	15	14	15	17	11	8	20
15	12	9	11	16	9	9	12
25	15	15	13	17	13	10	19
25	15	3	11	20	15	15	20
20	15	9	11	16	9	13	17
25	15	12	11	18	15	15	20
25	15	15	15	20	15	15	20
25	15	15	15	20	15	15	20
25	15	6	15	19	11	9	20
25	15	15	15	20	15	15	20
17	11	11	11	15	10	10	14
22	12	11	14	16	12	12	18
25	15	3	15	20	15	13	20
21	15	5	10	15	15	9	18
25	12	9	7	16	9	10	16
25	15	11	13	20	9	7	20
25	15	15	15	20	15	15	20
25	15	15	15	20	15	15	20
25	15	15	15	20	15	15	20
25	15	15	15	20	9	13	18
23	12	11	13	18	9	12	15
25	15	13	15	20	15	15	20
25	15	15	15	20	13	15	20
25	15	15	15	20	15	15	20
25	15	9	15	18	9	15	20
25	9	9	11	17	9	14	15
25	14	11	14	19	15	12	20
10	11	12	11	12	7	10	8
22	15	15	13	19	13	14	18
25	15	15	15	20	15	15	20
20	12	12	12	16	12	12	16
24	15	9	10	17	15	15	20
25	15	15	15	20	15	15	20
25	15	15	15	20	15	15	20
25	15	12	13	20	15	12	20
20	15	13	15	18	12	13	15
20	15	8	14	20	7	11	19
15	9	9	9	12	9	9	12
25	15	15	15	20	15	15	20
25	15	15	15	20	15	15	20
21	15	9	13	20	15	13	20
24	9	10	10	14	6	13	15
25	15	12	15	20	15	15	20
15	9	9	9	12	9	11	12
25	15	15	15	20	15	15	20

20	12	15	15	20	10	9	20
24	12	10	12	16	11	14	17
18	14	12	14	17	4	11	15
20	15	9	15	20	9	11	16
18	9	6	6	9	9	7	12
20	12	10	13	18	9	10	16
15	9	9	9	12	9	9	12
25	15	15	15	20	15	15	20
25	15	12	9	13	15	15	20
23	11	6	13	11	9	12	15
23	9	12	11	16	9	10	15
20	15	12	12	20	15	15	19
20	12	15	15	20	12	12	16
25	12	15	15	20	12	13	20
25	15	15	15	20	15	15	20
15	12	9	9	12	9	9	12
25	15	6	10	20	15	15	20
25	15	12	10	19	14	15	20
19	9	12	15	20	9	15	13
25	15	15	13	18	9	13	20
25	15	15	15	20	9	11	20
12	3	9	12	12	12	5	8
21	6	15	15	20	5	8	10
20	15	15	14	20	10	10	16
25	15	13	14	20	15	13	20
25	13	14	10	20	10	9	16
25	15	15	15	20	15	15	20
25	15	15	15	20	15	15	20
25	15	15	15	20	15	15	20
25	15	9	10	12	15	15	20
25	15	15	15	20	15	15	20
25	15	15	15	20	9	9	18
25	15	13	12	20	9	8	17
25	15	15	15	20	15	11	20
20	12	12	12	16	12	12	16
20	12	14	12	20	13	8	17
24	15	15	15	20	3	6	14
21	13	9	9	12	10	10	16
25	15	15	15	20	15	15	20
15	9	15	15	20	9	9	12
23	10	12	15	20	10	11	18
25	15	15	15	20	15	15	20
20	10	12	12	16	10	11	16
14	9	9	9	11	6	11	11
25	15	3	8	14	6	9	20
25	15	11	15	20	15	8	20
25	15	9	9	19	12	13	20
25	12	12	10	13	10	13	17
15	9	9	9	12	9	9	12
25	15	15	15	20	15	15	20
21	12	12	14	20	12	12	16
25	15	15	15	20	15	13	20
25	15	15	15	20	15	15	20
25	15	15	15	20	15	15	20

23	15	13	15	20	13	11	20
18	14	14	13	18	8	9	15
25	15	9	11	15	15	15	20
25	15	15	15	20	15	15	20
25	12	8	12	16	9	12	16
25	15	9	15	20	15	15	20
25	15	15	14	12	12	12	20
25	12	15	15	20	11	10	19
25	15	15	15	20	15	15	20
25	15	15	15	20	15	15	20
25	15	15	15	20	15	15	20
20	12	12	12	16	12	12	16
25	15	15	15	20	15	15	20
25	15	15	15	20	15	15	20
20	9	8	11	12	9	10	12
23	12	9	10	16	12	12	16
17	9	10	11	12	11	11	18
25	15	15	15	20	15	15	20
25	15	15	15	20	15	15	20
25	15	8	15	20	4	8	20
25	15	15	15	20	15	15	20
25	15	15	15	20	15	15	20
25	15	15	12	18	15	15	20
21	14	3	7	12	3	6	11
25	15	10	15	17	15	7	16
25	15	8	15	16	6	8	16
25	15	13	12	18	6	5	15
16	12	9	7	10	9	10	12
25	15	15	15	20	15	15	20
25	15	12	15	20	10	10	20
25	15	11	12	17	8	11	18
25	15	12	15	20	15	15	20
25	15	15	15	20	15	15	20
25	15	12	13	20	12	10	19
15	12	12	6	8	9	12	12
25	15	15	15	18	12	9	20
25	15	15	15	20	15	15	20
15	9	15	15	17	6	9	12
25	14	6	14	18	12	9	20
18	11	9	13	13	9	14	11
19	9	15	13	20	6	9	12
20	10	12	12	12	9	10	13
25	12	9	9	12	6	9	16
16	9	7	12	11	9	11	13
25	15	12	12	15	9	10	17
20	12	9	9	12	12	12	16
22	15	7	8	15	6	4	13
20	12	12	12	16	12	12	16
25	15	15	15	20	15	15	20
25	15	15	15	20	15	15	20
24	12	15	15	20	12	12	19
18	12	11	9	16	11	11	14
16	10	9	11	15	12	11	13
25	15	15	15	20	15	15	20

25	15	12	12	20	9	9	19
19	15	13	15	18	5	9	10
20	12	14	11	15	9	9	17
17	11	8	9	14	6	13	9
19	15	11	9	13	11	13	14
11	6	9	3	4	3	9	7
25	12	14	13	14	9	13	12
25	15	14	15	20	13	13	17
17	12	6	7	8	3	12	10
15	9	9	9	12	3	13	12
25	15	15	15	20	15	15	20
20	15	12	14	19	11	11	15
25	15	15	15	20	15	15	20
25	15	15	15	20	15	15	20
15	15	9	11	15	8	9	12
25	15	15	15	20	9	9	13
25	15	15	15	20	15	15	20
25	15	15	15	20	15	15	20
5	3	15	15	20	3	3	4
19	12	9	12	15	12	12	13
25	15	15	15	20	15	15	20
25	15	15	15	20	9	13	20
25	15	15	15	20	15	15	20
22	12	15	13	16	9	13	16
24	14	15	14	16	14	13	20
25	15	15	15	20	15	15	20
11	14	6	8	10	3	10	8
25	15	10	12	20	15	15	20
25	15	15	15	18	15	6	20
